# Two-dimensional (n = 1) ferroelectric film solar cells

**DOI:** 10.1093/nsr/nwad061

**Published:** 2023-03-07

**Authors:** Chen Wang, Jiahao Gu, Jun Li, Jianyu Cai, Lutao Li, Junjie Yao, Zheng Lu, Xiaohan Wang, Guifu Zou

**Affiliations:** College of Energy, Soochow Institute for Energy and Materials Innovations, and Key Laboratory of Advanced Carbon Materials and Wearable Energy Technologies of Jiangsu Province, Soochow University, Suzhou 215000, China; College of Mechanical and Electronic Engineering, Shandong University of Science and Technology, Qingdao 266590, China; College of Energy, Soochow Institute for Energy and Materials Innovations, and Key Laboratory of Advanced Carbon Materials and Wearable Energy Technologies of Jiangsu Province, Soochow University, Suzhou 215000, China; College of Energy, Soochow Institute for Energy and Materials Innovations, and Key Laboratory of Advanced Carbon Materials and Wearable Energy Technologies of Jiangsu Province, Soochow University, Suzhou 215000, China; College of Energy, Soochow Institute for Energy and Materials Innovations, and Key Laboratory of Advanced Carbon Materials and Wearable Energy Technologies of Jiangsu Province, Soochow University, Suzhou 215000, China; College of Energy, Soochow Institute for Energy and Materials Innovations, and Key Laboratory of Advanced Carbon Materials and Wearable Energy Technologies of Jiangsu Province, Soochow University, Suzhou 215000, China; College of Energy, Soochow Institute for Energy and Materials Innovations, and Key Laboratory of Advanced Carbon Materials and Wearable Energy Technologies of Jiangsu Province, Soochow University, Suzhou 215000, China; College of Energy, Soochow Institute for Energy and Materials Innovations, and Key Laboratory of Advanced Carbon Materials and Wearable Energy Technologies of Jiangsu Province, Soochow University, Suzhou 215000, China; College of Energy, Soochow Institute for Energy and Materials Innovations, and Key Laboratory of Advanced Carbon Materials and Wearable Energy Technologies of Jiangsu Province, Soochow University, Suzhou 215000, China; College of Energy, Soochow Institute for Energy and Materials Innovations, and Key Laboratory of Advanced Carbon Materials and Wearable Energy Technologies of Jiangsu Province, Soochow University, Suzhou 215000, China

**Keywords:** molecular ferroelectric film, 2D perovskite ferroelectric film, multiaxial ferroelectrics, out-of-plane charge transport, solar cell

## Abstract

Molecular ferroelectrics that have excellent ferroelectric properties, a low processing temperature, narrow bandgap, and which are lightweight, have shown great potential in the photovoltaic field. However, two-dimensional (2D) perovskite solar cells with high tunability, excellent photo-physical properties and superior long-term stability are limited by poor out-of-plane conductivity from intrinsic multi-quantum-well electronic structures. This work uses 2D molecular ferroelectric film as the absorbing layer to break the limit of multiple quantum wells. Our 2D ferroelectric solar cells achieve the highest open-circuit voltage (1.29 V) and the best efficiency (3.71%) among the 2D (n = 1) Ruddlesden–Popper perovskite solar cells due to the enhanced out-of-plane charge transport induced by molecular ferroelectrics with a strong saturation polarization, high Curie temperature and multiaxial characteristics. This work aims to break the inefficient out-of-plane charge transport caused by the limit of the multi-quantum-well electronic structure and improve the efficiency of 2D ferroelectric solar cells.

## Teaser

Two-dimensional molecular ferroelectric film solar cells are fabricated to break the inefficient out-of-plane charge transport from the limit of multi-quantum well electronic structure for the efficiency of 2D ferroelectric solar cells.

## INTRODUCTION

Ferroelectrics with effective out-of-plane charge separation have attracted renewed attention [1–4]. Over the past decade, ferroelectric photovoltaic devices have facilitated great progress in the areas of anomalous photovoltaic effects [5–7], interface engineering [8,9] and single and multilayer solar cells [10–12]. As a cutting-edge topic related to ferroelectrics, a series of molecular ferroelectrics with excellent ferroelectric attributes (e.g. multiaxial [13], large spontaneous polarization [14] and high Curie temperature [15]), along with the properties of low annealing temperature [16], narrow bandgap [17,18] and mechanical flexibility, has been reported [19,20]. Exploring molecular ferroelectrics for efficient photovoltaic devices is highly promising.

**Figure 1.**
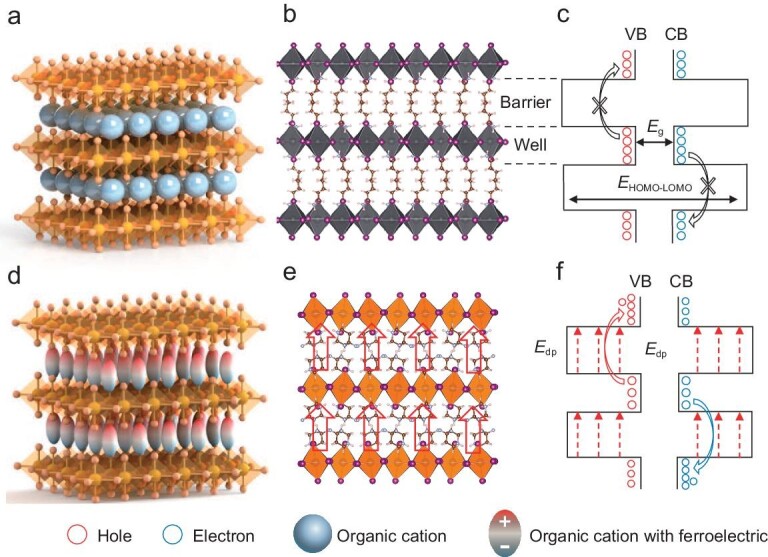
Schematic diagram of the poor out-of-plane charge transport in 2D perovskites and enhanced out-of-plane charge transport in the 2D molecular ferroelectrics. (a) Schematic diagram of the 2D perovskite structure. (b) Crystal structure of the 2D perovskites, with (PEA)_2_PbI_4_ as an example. (c) MQW structures and poor charge transport in 2D perovskites. (d) Schematic diagram of the 2D molecular ferroelectric structure: the ferroelectricity originates from the directional arrangement of organic cations. (e) Crystal structure of the 2D molecular ferroelectric (DFPD)_2_PbI_4_: the arrow represents the depolarization field. (f) Enhanced charge transport in the MQWs under the influence of ferroelectricity.

Two-dimensional (2D) perovskites have the significant advantage of stability for next-generation photovoltaic semiconductors [21–23]. As is well known, the inferior out-of-plane charge transport caused by the insulating organic spacer layers is the biggest obstacle for photovoltaic performance in 2D perovskite solar cells (PSCs) [24]. As shown in Fig. 1a, 2D (n = 1) perovskites are composed of an alternating arrangement of inorganic frameworks and organic spacer layers that make the crystal growth direction parallel to the substrate (Fig. 1b). The dramatically contrasting dielectric constant between inorganic layers and organic spacer layers forms a multiple quantum well (MQW) electronic structure, which results in optoelectronic anisotropy and poor out-of-plane conductivity between inorganic layers (Fig. 1c). In recent years, much progress has been made to ensure efficient charge transport, including out-of-plane orientation [25,26] and organic cation optimization [27,28]. For now, the performance of 2D (n = 1, where n is the number of inorganic sheets placed between each organic layer) PSCs remains unsatisfactory [26,29]. Thus, finding effective strategies to promote the out-of-plane charge transport of 2D perovskites is desirable.

**Figure 2.**
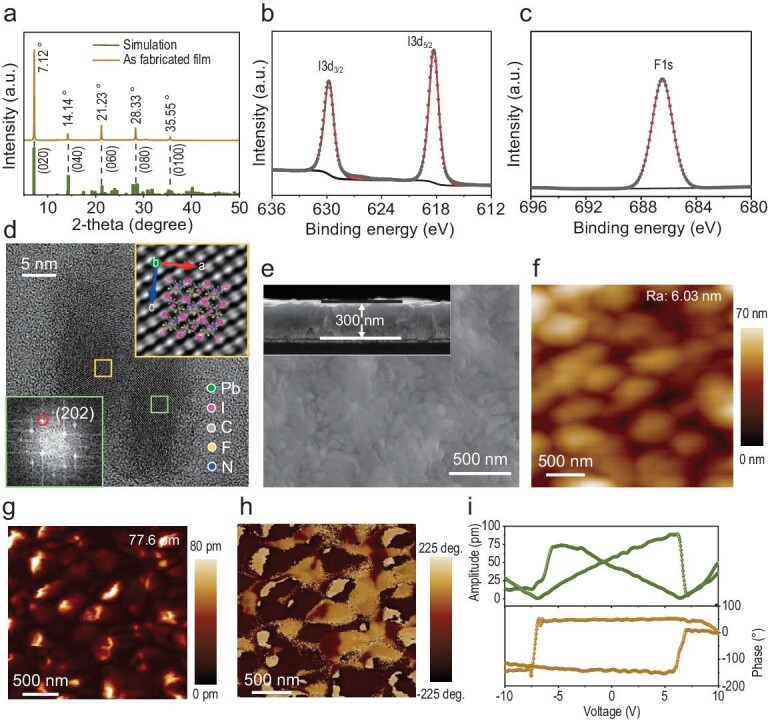
Characterization of the (DFPD)_2_PbI_4_ film. (a) XRD pattern. XPS of (b) I 3d and (c) F 1 s. (d) HRTEM image. (e) SEM and cross-section image. (f) AFM image of the tested area. (g) Amplitude and (h) phase images from the PFM. (i) Hysteretic dependence of the PFM phase and amplitude with the applied DC bias.

2D molecular ferroelectrics are generally considered strict single-layer (n = 1) materials. The key point is that these molecular ferroelectrics have both 2D structures and ferroelectricity (Fig. 1d). Recently, H/F substitution on the organic cations has been proposed as an effective method for designing and modifying the properties of 2D molecular ferroelectrics [16,19]. The (4,4-difluoropiperidinium)_2_PbI_4_ ((DFPD)_2_PbI_4_) was designed by the hydrogen/fluorine (H/F) substitution on the organic cations [14]. After difluorination on the organic cation of non-ferroelectric (piperidinium)PbI_3_, the obtained (DFPD)_2_PbI_4_ shows clear ferroelectricity. Therefore, it is of great importance to explore the application of molecular ferroelectric (DFPD)_2_PbI_4_ composed of DFPD and lead iodide in the photovoltaic field. This work uses a 2D molecular ferroelectric (DFPD)_2_PbI_4_ with multiple polar axes, large spontaneous polarization (10 C/cm^2^), high Curie temperature (373 K) and narrow bandgap as the absorbing layer for fabricating 2D ferroelectric perovskite solar cells (FSCs). Ascribed to the fascinating ferroelectricity arising from the order–disorder transition of organic cations (Fig. 1e) [14], an additional depolarization field will optimize the poor out-of-plane charge transport between the inorganic layers (Fig. 1f). Benefiting from this characteristic, the fabricated 2D FSCs achieved the highest open-circuit voltage (1.29 V) and the best efficiency (3.71%) in 2D (n = 1) Ruddlesden–Popper PSCs. Compared with the 2D non-ferroelectric perovskites, ferroelectricity has a vital influence on film optoelectronic properties and directly enhances the out-of-plane charge transport. This work shows that ferroelectricity can promote the out-of-plane charge transport between inorganic layers and demonstrates a promising application for 2D molecular ferroelectrics.

## RESULTS AND DISCUSSION

High-quality thin films are conducive to fabricating high-performance solar cells. Herein, 2D molecular ferroelectric (DFPD)_2_PbI_4_ was prepared through a low-temperature chemical solution deposition method. (DFPD)_2_PbI_4_ shows a 2D hybrid perovskite structure. The difluorinated DFPD organic cations form a 2D organic network through C–H···F–C interactions (Fig. S1), which is in favor of building and stabilizing the 2D PbI_4_^2–^ framework [30]. X-ray diffraction (XRD), X-ray photoelectron spectroscopy (XPS), high-resolution transmission electron microscopy (HRTEM), scanning electron microscopy (SEM), ultraviolet-visible spectroscopy (UV-Vis), atomic force microscopy (AFM) and piezoresponse force microscopy (PFM) were used to characterize the crystal structure, topography, optical properties and ferroelectricity of as-grown films. As is well known, films with a low-temperature phase have a polar space group, Aba2, with a point group, mm2 [14]. The XRD results (Fig. 2a) revealed that the characteristic peaks had high intensity, small full width at half maximum, obvious orientation and no other impurity phases (e.g. PbI2). The peak positions at 7.12°, 14.14°, 21.23°, 28.33° and 35.55° corresponded to the (020), (040), (060), (080) and (0100) crystallographic planes, respectively, indicating the in-plane orientation (010) of the as-grown films. The XPS result of the (DFPD)_2_PbI_4_ film was recorded to investigate the surface chemical properties. In Fig. 2b, 329.75 and 618.28 eV indicate the peaks of I 3d3/2 and I 3d5/2, respectively, which came from the PbI_6_ octahedral layers. A single symmetry peak at 686.45 eV denoted the F 1s that came from the fluoropiperidine cations (Fig. 2c). The HRTEM result is presented in Fig. 2d to investigate the crystal microstructure of the as-grown films. The HRTEM image presented a clear lattice structure suggesting a high crystallinity. The illustration with a yellow border is the processed TEM lattice fringe of the area marked with a yellow square, which corresponds to the (DFPD)_2_PbI_4_ crystalline structure from the b-direction projection view. The fast Fourier transform image processed from the area marked by a green square had (202) planes with an interplanar spacing of 3.27 Å. The highly ordered lattice fringes matched well with the corner-sharing inorganic octahedral framework, indicating that the films were highly orientated along the in-plane growth, which was consistent with the XRD results. The SEM images (Fig. 2e) showed that the film surface was continuous and dense. The cross-section image revealed that the film had good contact with the substrate, and no pinhole defects existed. Different from oxide ferroelectrics with a wide bandgap (>3 eV) [31], the as-grown 2D molecular ferroelectric film showed a narrow bandgap of 2.32 eV (Fig. S2). A narrow and suitable bandgap is helpful in achieving effective light absorption in an absorbing layer. Note that an obvious correlation can be found between the bandgap and the thickness. Depending on the film thickness, the bandgap can be adjusted from 2.24 to 2.34 eV (Fig. S3). This thickness-dependent bandgap is always found in van der Waals layered structures, which is often attributed to the prevailing mechanism of energy splitting of band edges caused by the interlayer hopping [32,33]. PFM was performed to evaluate the out-of-plane ferroelectric properties. Figure 2f shows the AFM image of the test area. The film roughness was calculated as 6.03 nm, which benefited the interfacial contact and reduced the surface defects. The PFM amplitude and phase images in Fig. 2g and h illustrate excellent ferroelectricity. The various angles between the domain walls signified the potential presence of non-180° domains, indicating that (DFPD)_2_PbI_4_ was a multiaxial ferroelectric [15]. The as-obtained PFM amplitude and the phase signal showed no crosstalk of the piezoelectric signals with topography, suggesting that the PFM contrast came from the domains instead of the surface roughness [30]. Polarization switching is an evidence of ferroelectricity. The local PFM spectrum showed distinct 180° switching of the PFM phase, as well as a strong hysteresis and a butterfly-like shape in the amplitude under the direct-current bias voltages (Fig. 2i). This suggested a typical ferroelectric polarization switching process and demonstrated the ferroelectric properties of the as-grown films. Differential scanning calorimetry and thermo-gravimetric analysis (Fig. S4) were used to assess the ferroelectric stability. The decomposition and Curie temperatures were 451 K and 441 K, respectively, indicating the occurrence of the ferroelectric phase transition at a Curie temperature of 441 K and excellent stability of the ferroelectricity at room temperature.

**Figure 3.**
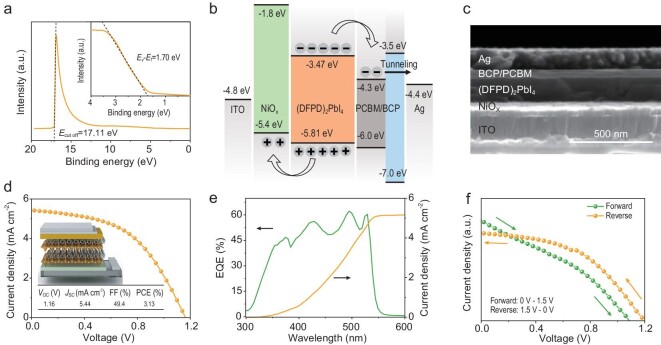
Characterization of the 2D FSCs. (a) Ultraviolet photoelectron spectroscopy of the as-grown films. (b) Schematic diagram of the device band structure. (c) Cross-section image. (d) *J*–*V* curve under AM 1.5 G illumination and schematic diagram of the device. (e) External quantum efficiency (EQE) measurement. (f) *J*–*V* curve under different scanning directions (forward and reverse).

In Fig. 3a, ultraviolet photoelectron spectroscopy was performed to optimize the devices with suitable band structures and an effective charge transport. The Fermi level (*E*_F_), bottom of the conduction band (*E*_C_), and top of the valence band (*E*_V_) were estimated as −4.11, −3.47 and −5.81 eV, respectively. Figure S5 shows the band diagram. (DFPD)_2_PbI_4_ was found as an n-type semiconductor. An inverted structure was adopted with indium tin oxide (ITO) and silver as the transparent conductive oxide and the top electrode, respectively, to reduce the Schottky barrier between the absorbing layer and the electrode. Considering the band structure and the preparation conditions (Fig. S6), NiOx and [6,6]-phenyl-C61-butyric acid methyl ester (PCBM) were selected as the hole and electron transport layers, respectively. Bathocuproine (BCP) was used as a hole-blocking layer that could reduce the interface recombination loss and improve the device performance [34]. Figure 3b and c illustrate the band structure and the cross-section image of the device, respectively. The rainbow-like band structure was beneficial to reducing the recombination loss, promoting the charge separation and enhancing the device performance. Figure 3d shows that the as-fabricated 2D FSCs have an efficiency of 3.13%, which is higher than that of the bandgap-similar non-ferroelectric 2D (n = 1) PSCs [26]. The device's open circuit voltage (*V*_OC_), short-circuit current (*J*_SC_) and fill factor (FF) were 1.16 V, 5.44 mA cm^−2^ and 49.4%, respectively. Statistics on the performance of 38 devices are collected in Fig. S7. The effect of the BCP layer on device performance is discussed in Fig. S8. Table S1 shows the performance summary of the 2D (n = 1) Ruddlesden–Popper PSCs tested by a sun simulator. Figure S9a and b present the plots of *V*_OC_ and power conversion efficiency (PCE) versus the bandgap. Figure 3e illustrates the external quantum efficiency (EQE) measurement of the 2D FSCs. The device exhibited a continuous photoresponse from 300 to 550 nm with an EQE of ∼61% at 529 nm. The *J*_SC_ calculated from the EQE was ∼5.16 mA cm^−2^, which was slightly lower than the value observed in the *J*–*V* curves. Figure 3f shows the abnormal hysteresis in the 2D FSC. This difference between the forward and reverse sweep could be caused by ferroelectricity [35].

**Figure 4.**
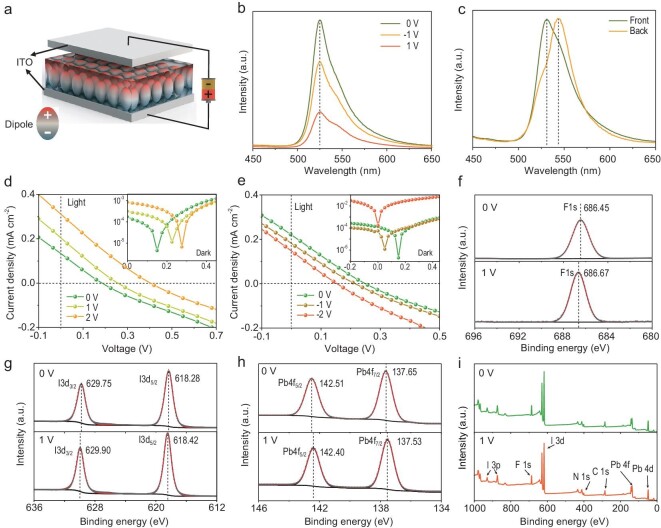
Characterization of the (DFPD)_2_PbI_4_ films before and after polarization. (a) Schematic diagram of the polarization. (b) Steady-state PL of the films with different poling conditions. (c) Dual-emission peaks of the (DFPD)_2_PbI_4_ film excited from the front and back of the sample. *J*–*V* curves of the (DFPD)_2_PbI_4_ devices (without transport layers) with (d) positive and (e) reverse poling. The illustrations show the corresponding dark current. XPS results of (f) F 1 s, (g) I 3d and (h) Pb 4f and (i) survey of the (DFPD)_2_PbI_4_ film before and after polarization.

Ferroelectrics have a distinct feature with a unique polarization orientation, which should have a crucial influence on the film's optoelectronic properties [36,37]. Figure 4a is a schematic diagram of the film polarization. The film spin-coated on ITO is in close contact with another independent electrode, ensuring effective polarization. Figure 4b shows the steady-state photoluminescence (PL) data with bias voltages of 0, −1 and 1 V. Compared with the non-polarized film, the PL intensity decreased after polarization, suggesting that the internal radiation recombination was suppressed. The decrease in PL intensity was caused by the influence of the depolarization field, which resulted in the effective separation of the electrons and holes instead of a radiative recombination. The PL data of the non-ferroelectric (PEA)_2_PbI_4_ under the same polarization conditions were collected to further confirm this conclusion (Fig. S10). No quenching was observed in the PL before and after polarization. Note that asymmetric PL peaks existed in the as-prepared (DFPD)_2_PbI_4_ and (PEA)_2_PbI_4_ film (Fig. S10a and b). These asymmetric PL peaks were due to several reasons, including two kinds of lattice phases [38] and dual bandgap [39]. We performed the XRD on the log scale to determine the possible presence of different phases in the as-grown (DFPD)_2_PbI_4_ film. In Fig. S10c, no different phases (e.g. n > 1 2D perovskite) or excess raw material were found. In addition, 2D perovskites showed dual-emission peaks arising from the surface and interior of the material [39]. We further explored the asymmetric peaks by comparing the PL curves with different incidence angles (front and back, Fig. S11a). When excited from the front, the PL showed dual-emission peaks with a strong peak appearing at 530 nm (Fig. S11b). When excited from the back, dual-emission peaks were still observed, but the peak intensity at 530 nm was weakened, and the strong peak appeared near 543 nm (Fig. 4c). The asymmetric PL peaks could be induced by the dual-emission peak phenomenon. A similar mechanism could be used to explain the two peaks in the PL of (PEA)_2_PbI_4_ (Fig. S11c). A further analysis of the PL in Fig. 4b revealed that the PL intensity decreased after the −1 V polarization, which was still twice as much as that after the +1 V polarization. This difference in the PL intensity could have been caused by the original field of the device itself (Fig. S12). The original device field could be generated by the spontaneous polarization in the (DFPD)_2_PbI_4_ film. A spontaneous polarization in the film is believed to promote out-of-plane conductivity, which makes the 2D FSCs perform better than other 2D PSCs. We fabricated photovoltaic devices without transport layers to explore the influence of ferroelectricity on the film photovoltaic response. The non-polarized device showed a small voltage of 0.19 V and a low photocurrent of 0.13 mA cm^−2^, which could be induced by the spontaneous polarization field and the Schottky barrier from the film/electrode interfaces (Fig. 4d). When positively polarized from 0 to 2 V, both the device voltage and the photocurrent increased to 0.40 V and 0.31 mA cm^−2^, respectively. The voltage increased by 0.21 V, and the dark current revealed an increase in the built-in electric field. The reverse polarization was the complete opposite (Fig. 4e). The voltage after the reverse polarization dropped by 0.10 V, as the reverse bias voltage increased from 0 to −2 V. Figure S13 shows an excessive bias voltage (>2 V) that will cause a short-circuiting of devices. XPS is a sensitive tool for evaluating the incommensurate modulation of the crystal structure in ferroelectrics [40]. The XPS results indicate that the elemental binding energy has been changed before and after polarization. As shown in Fig. 4f, the peak shape of F 1s, I 3d and Pb 4f did not change significantly before and after polarization, but the peak position shifted slightly. The F 1s peak red-shifts from 686.45 eV to 686.67 eV, an increase of 0.22 eV. This peak shift, without new features, indicates changes in the chemical environment of the F atom, which could be attributed to the order–disorder transition of the organic cation [41]. The same increase in binding energy also occurs on the spin-orbit doublet of I 3d (Fig. 4g). The binding energy of I 3d_3/2_ and I 3d_5/2_ increased from 629.75 eV and 618.28 eV to 629.90 eV and 618.42 eV, respectively. This suggests a lower negative partial charge on I atom after polarization, which could be due to the longer bond lengths at bias voltages [42]. On the other hand, the binding energy of Pb 4f decreased after polarization. The binding energy of Pb 4f_5/2_ and Pb 4f_7/2_ at 142.51 eV and 137.65 eV decreases to 142.40 eV and 137.53 eV (Fig. 4h). These shifts in binding energy indicate that the lead iodide inorganic framework could also participate in the polarization process. Considering the use of a silver electrode for polarization, silver migration during polarization may be responsible for the change in the element binding energy [43]. However, we did not find evidence of silver migration in the survey (Fig. 4i).

**Figure 5.**
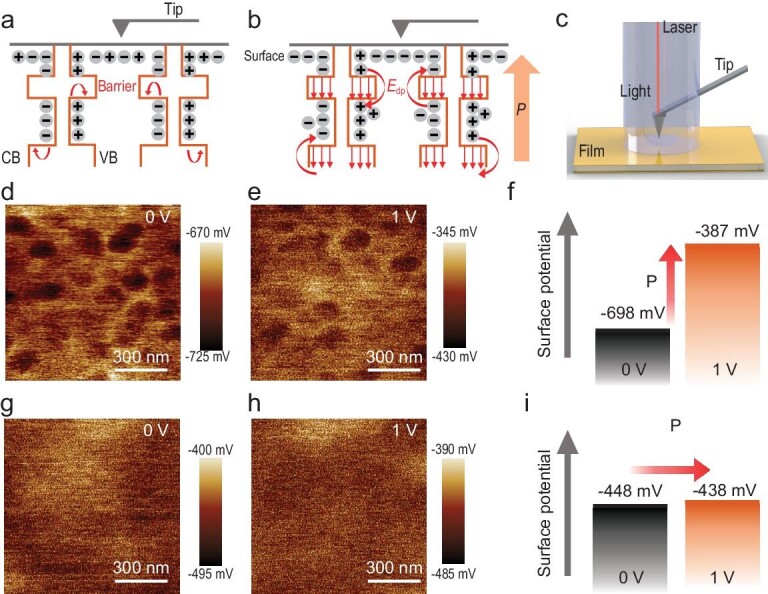
KPFM of the 2D perovskite and 2D molecular ferroelectric films. Schematic diagram of the charge transport in the (a) 2D perovskite and (b) 2D molecular ferroelectrics. The orange and red straight arrows represent the orientation of the external polarization and depolarization fields, respectively. (c) Diagram of the KPFM test. Surface potential images of the (DFPD)_2_PbI_4_ film (d) before and (e) after polarization. (f) Mean surface potential of the (DFPD)_2_PbI_4_ film before (0 V) and after (1 V) polarization. Surface potential images of the (PEA)_2_PbI_4_ film (g) before and (h) after polarization. (i) Mean surface potential of the 2D perovskite (PEA)_2_PbI_4_ before (0 V) and after (1 V) polarization. (All images share the same scale bar.)

The hypotheses in Fig. 5a and b were proposed to explain the ferroelectric mechanism in the 2D FSCs. For the 2D perovskites, the existence of potential wells (inorganic layer) and barriers (organic layer) meant that the photo-generated charges tended to be in an in-plane transport, which led to a serious charge recombination and consequently a limited performance (Fig. 5a) [24,44]. Nevertheless, the existence of ferroelectricity could break the barrier effect of the MQWs on out-of-plane charge transport. Figure 5b illustrates that the existence of the depolarization field can additionally promote the electron and hole transport. When the depolarization field points out of the plane, the electrons and holes bound in the MQWs have a high possibility of being transported between inorganic layers. This process can be investigated through Kelvin probe force microscopy (KPFM) (Fig. 5c). The surface potential of the (DFPD)_2_PbI_4_ film (Film 1) without polarization was collected and presented in Fig. 5d. The mean potential was calculated as −698 mV. Figure 5e depicts the surface potential image of Film 1 with a mean value of −387 mV after polarization with a 1 V bias voltage for 300 s. Figure 5f indicates that the mean surface potential of Film 1 before and after polarization was reduced by −311 mV. This change in the surface potential can be induced by the charge accumulation on the surface during polarization or the photo-generated charge transport to the surface under the depolarization field promotion. We further studied the role of ferroelectricity by performing KPFM on the non-ferroelectric 2D perovskite (PEA)_2_PbI_4_ (Film 2) under the same conditions. Figure 5g and h show the surface potential of Film 2 before and after polarization. Different from that of Film 1, the mean surface potential of Film 2 remained nearly the same during polarization. Topographic images for the PFM test of Films 1 and 2 are shown in Fig. S14. As shown in Fig. 5i, the mean surface potentials before and after polarization were −448 and −438 mV, respectively, with only a −10 mV drop. In conclusion, the obvious surface potential change is associated with ferroelectricity, and the existence of ferroelectricity plays an important role in promoting the out-of-plane charge transport in 2D solar cells. An electrochemical impedance spectroscopy analysis was performed under dark conditions to further demonstrate the role of ferroelectricity on the charge transport (Fig. S15). Therefore, ferroelectricity has a positive effect on reducing the electron–hole recombination loss, promoting carrier separation and improving the performance of 2D FSCs.

**Figure 6.**
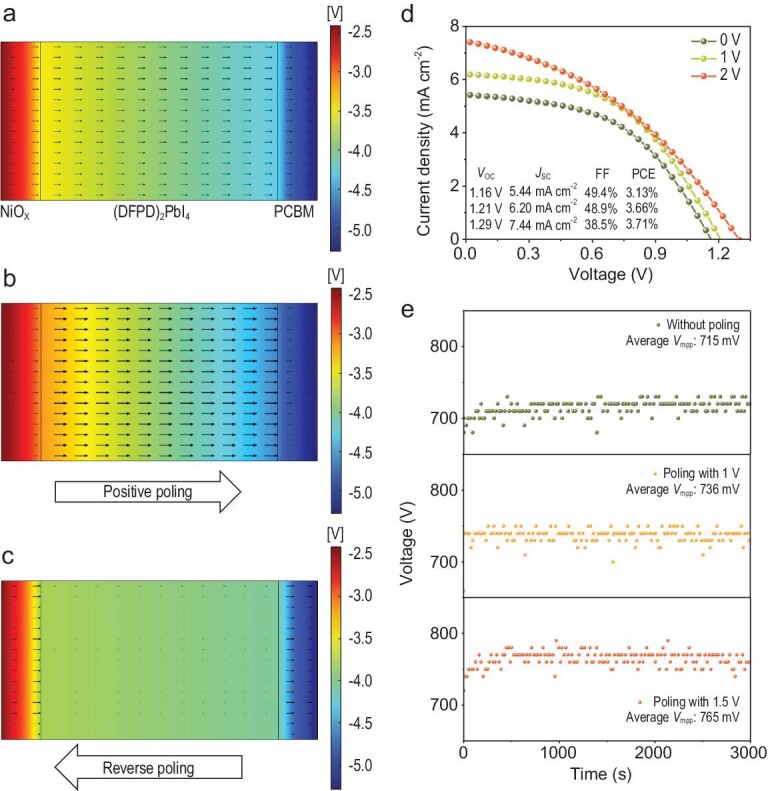
Simulation and photovoltaic characterization of the 2D FSCs. Electric field intensity of the (a) pristine state, (b) positive polarization and (c) reverse polarization. (d) *J*–*V* curves of the 2D FSC after positive poling. (e) Field stability analysis with bias voltages of 0, 1 and 1.5 V.

In general, ferroelectrics with a depolarization field in an appropriate direction can further improve device performance. We performed simulations of the ferroelectric photovoltaic devices using COMSOL to understand the space charge region and the electric potential in the p–i–n junction under the influence of polarization [45,46]. The geometric model adopted the same p–i–n structure as a 2D (DFPD)_2_PbI_4_ solar cell: NiO_X_ (50 nm)/film (300 nm)/PCBM (50 nm). Table S2 lists the material parameters of the simulations. The depolarization field strength was simulated by changing the surface charge density [46]. First, the depletion region can be modulated by polarization (Fig. S16). Compared with the pristine state (without polarization), the width of the depletion region between the active and transport layers decreased after positive polarization from NiO_X_ to PCBM. In contrast, the depolarization field after the reverse polarization (from PCBM to NiO_X_) elongated the width of the depletion region. The effect of polarization on the carrier transport was also investigated by analyzing the potential distribution. Figure 6a shows the carrier transport in the pristine state affected only by the built-in electric field from the heterojunction. The direction and the size of the arrows indicate the electron flow and the intensity of the electric field at the point, respectively. Figure 6b shows that the carrier transport was enhanced under positive polarization. This enhanced electric field will reduce recombination and subsequently improve the *V*_OC_ and the PCE. For comparison, Fig. 6c shows the potential distribution after reverse polarization. The carrier transport inside the material was gradually suppressed as the reverse depolarization field increased (Fig. S16). Figure 6d presents device performance by positive poling at 1 and 2 V. The *V*_OC_ increased from 1.16 to 1.29 V, with the highest *V*_OC_ in the 2D (n = 1) PSCs. Accordingly, the PCE increased to 3.71%, which was the best efficiency among the 2D (n = 1) Ruddlesden–Popper PSCs. These enhancements in the *V*_OC_ and PCE revealed the role of ferroelectricity in device optimization. For comparison, the 2D (PEA)_2_PbI_4_ PSCs were evaluated under the same conditions in Fig. S17. We observed almost no change before and after polarization. Maximum power point tracking was used to investigate the field stability of the (DFPD)_2_PbI_4_ FSCs. Figure 6e shows the *V*_MPPT_ of the (DFPD)_2_PbI_4_ FSCs after polarization. The average *V*_MPPT_ of the device without poling was 715 mV. After poling with 1 V, the average *V*_MPPT_ increased to 736 mV and remained stable. Device poling with a higher bias of 1.5 V showed a higher and stable *V*_MPPT_ of 765 mV. The device *V*_MPPT_ showed a slight decay when the bias voltage increased to 2 V (Fig. S18). This voltage decay was more pronounced at a higher bias (2.5 V) polarization. This device performance degradation may be due to the damage caused by the excessive bias voltage, as described in Fig. S19. Overall, device performance can be steadily improved under an appropriate bias voltage.

## CONCLUSION

This work fabricated a new kind of 2D FSC to solve the poor out-of-plane charge transport of 2D PSCs. Due to the excellent ferroelectricity, the charges trapped in the organic layers were effectively separated, transported and collected to achieve the highest open-circuit voltage (1.29 V) among reported 2D (n = 1) PSCs, and the best efficiency (3.71%) among 2D (n = 1) Ruddlesden–Popper PSCs. This work provides a new way for improving the inefficient out-of-plane charge transport of 2D solar cells and demonstrates a promising application for the 2D molecular materials used in optoelectronics. Molecular ferroelectrics have broad development prospects with regard to obtaining stable, high-voltage and efficient photovoltaic devices. Benefiting from excellent ferroelectric and photoelectric properties, molecular ferroelectrics have potential application in the field of photoelectrochemical hydrogen production.

## METHODS

### Materials

(DFPD)_2_PbI_4_ powder: 4, 4-difluoropiperidinium hydrochloride, PbI_2_ and hydriodic acid (57 wt% in water) were purchased from Tokyo Chemical Industry. Stoichiometric amounts of PbI_2_ (23 g, 50 mmol) and 4,4-difluoropiperidinium hydrochloride (15.8 g, 100 mmol) were dissolved in the hydriodic acid (250 mL) at 443 K, and the solution was rapidly cooled to obtain the powder sample of (DFPD)_2_PbI_4_.

### Device fabrication

A device structure of ITO/NiOx/absorber/PCBM/BCP/Ag was employed to prepare 2D ferroelectric PSCs. The ITO was washed first before the electron beam evaporated the NiOx, followed by spin coating of the absorber, PCBM and BCP. Finally, a 100 nm silver electrode was deposited by thermal evaporation. The parameters of the spin coating are detailed in the supporting information.

## Supplementary Material

nwad061_Supplemental_File
