# Supplementary material for: Two-dimensional (n = 1) ferroelectric film solar cells
Source: Natl Sci Rev. 2023 Mar 7;10(7):nwad061. doi: 10.1093/nsr/nwad061 (PMC10434298; doi:10.1093/nsr/nwad061)
Supplement: nwad061_Supplemental_File [file nwad061_supplemental_file.docx]

**Supporting information**

**Two-dimensional (n=1) Ferroelectric Film Solar Cells**

Chen Wang ^[a],[b]#^, Jiahao Gu ^[a]#^, Jun Li ^[a]^, Jianyu Cai ^[a]^, Lutao Li ^[a]^, Junjie Yao ^[a]^, Zheng Lu ^[a]^, Xiaohan Wang ^[a]^ and Guifu Zou ^[a]^

^[a]^ College of Energy, Soochow Institute for Energy and Materials Innovations, and Key Laboratory of Advanced Carbon Materials and Wearable Energy Technologies of Jiangsu Province, Soochow University, Suzhou, 215000, P. R. China.

^[b]^ College of Mechanical and Electronic Engineering, Shandong University of Science and Technology, Qingdao. 266590, P. R. China.

*Email: zouguifu@suda.edu.cn

**Experimental section**

**Film fabrication.** (DFPD)_2_PbI_4_ solutions with different concentrations were dissolved in a mixed solution of dimethylformamide and dimethyl sulfoxide, the solutions were stirred at 30°C for 3 hours to become clear. The obtained precursors were spin coated on ITO. The ITO was ultrasonic cleaning with acetone, ethanol and ultrapure water for 10 minutes in advance. The spin-coated films were immediately annealed at 100°C for 10 minutes. All processes were carried out in the glove box.

**Device fabrication.** ITO glass was purchased from Liaoning Youxuan New Energy Technology Co., Ltd. Nickel oxide was prepared by magnetron sputtering method. 20 mg/ml PCBM was spin coated at 2000 rpm for 40s, and let it stand for 40 minutes under vacuum to promote film formation. BCP with concentration of 0.4 mg/ml was spin coated at 4000 rpm for 15s. 100 nm silver electrode was prepared by thermal evaporation.

**Film and device characterization.** Thermogravimetric analysis was measured by TG/DTA 7300 from SII Nano Technology under an argon atmosphere. The X-ray diffraction (XRD) was taken on a D8 Advance of Bruker. The morphologies of the films were observed using a scios scanning electron microscope from FEI. An atomic force microscope (AFM) and Piezoresponse Force Microscopy (PFM) was used by Dimension Icon (Bruker). UV–vis spectra were tested on a Lambda 750S from PerkinElmer. The steady-state photoluminescence (PL) was obtained through Fluoromax-4 Spectrofluorometer from Horiba. Ultraviolet Photoelectron Spectrometer (UPS) were obtained using an ESCALAB 250Xi XPS. The current density voltage (*J*–*V*) data of devices with an area of 0.0725 cm^2^ were measured by an XES-70S1 solar simulator (SAN-E1) with 2400 source meter (KEITHLEY) under simulated air mass (AM) 1.5 solar spectrum illumination at 100 mW/cm^2^. The space-charge limited current (SCLC) was tested by 2400 source meter from KEITHLEY. The external quantum efficiency (EQE) was measured by QE-R3018 from Enlitech (Taiwan, China).


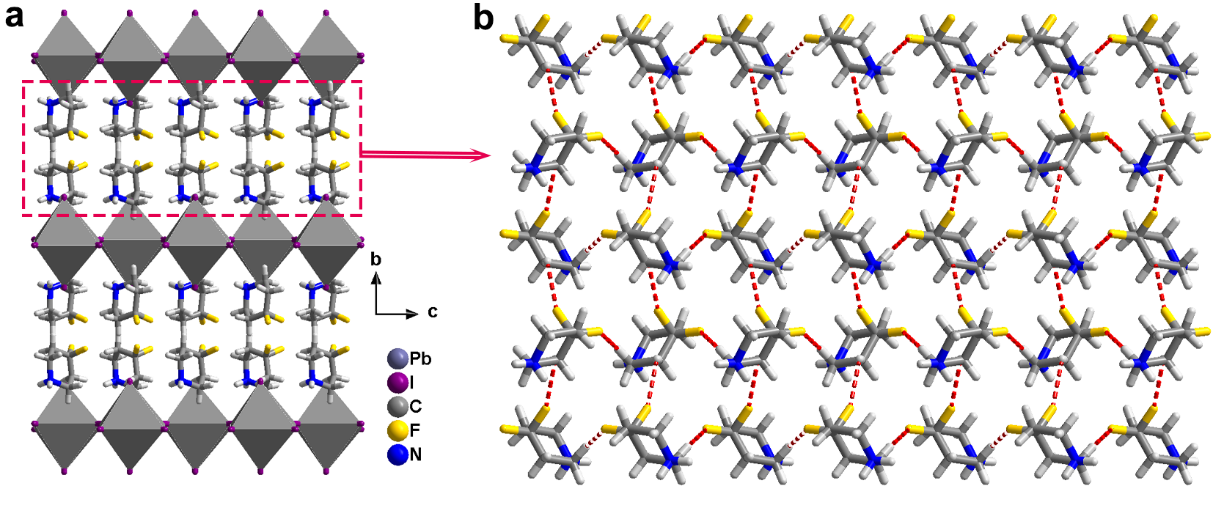


**Figure S1**. (a) 2D perovskite structure of (4, 4-difluoropiperidinium)_2_PbI_4_, (b) 4,4-difluoropiperidinium organic cation layer. The red dotted line represents weak C-H···F-C hydrogen-bonding interactions.


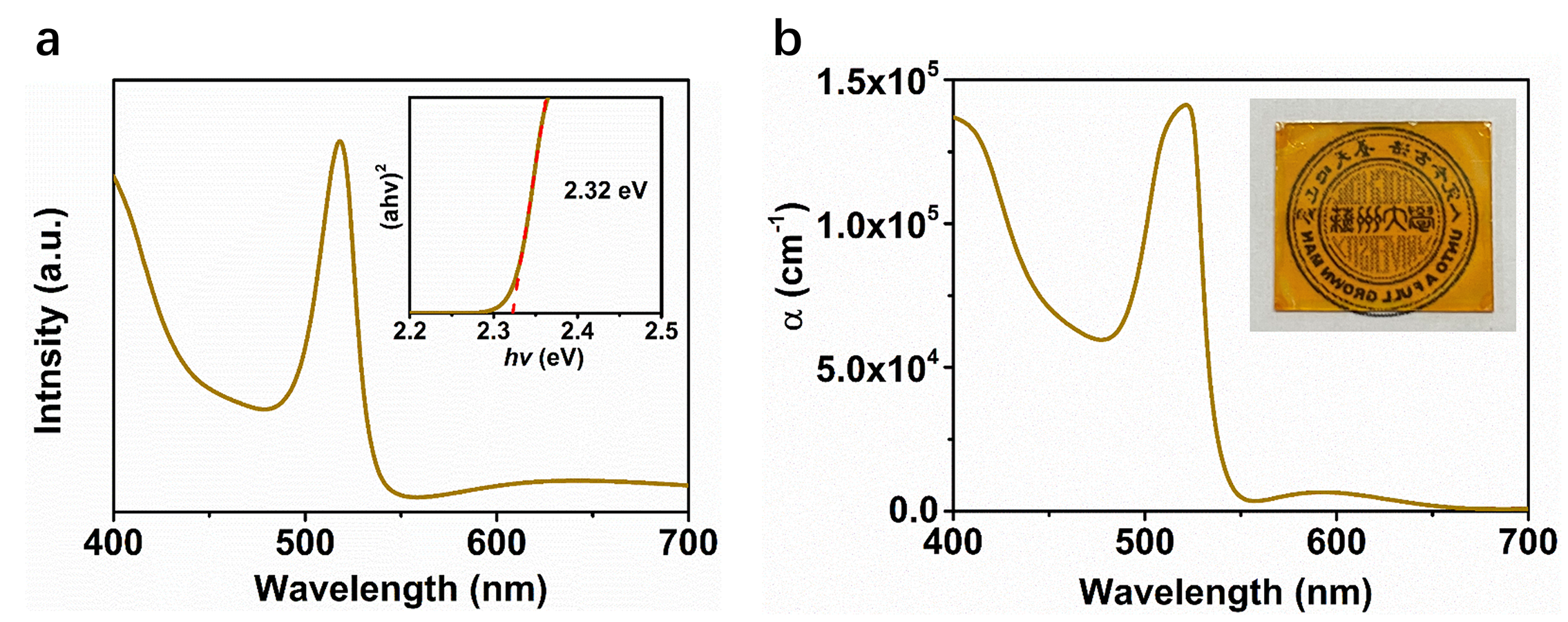


**Figure S2**. UV-Vis spectra of obtained film and the illustration is the plot of (*αhv*)^2^ versus *hv*.


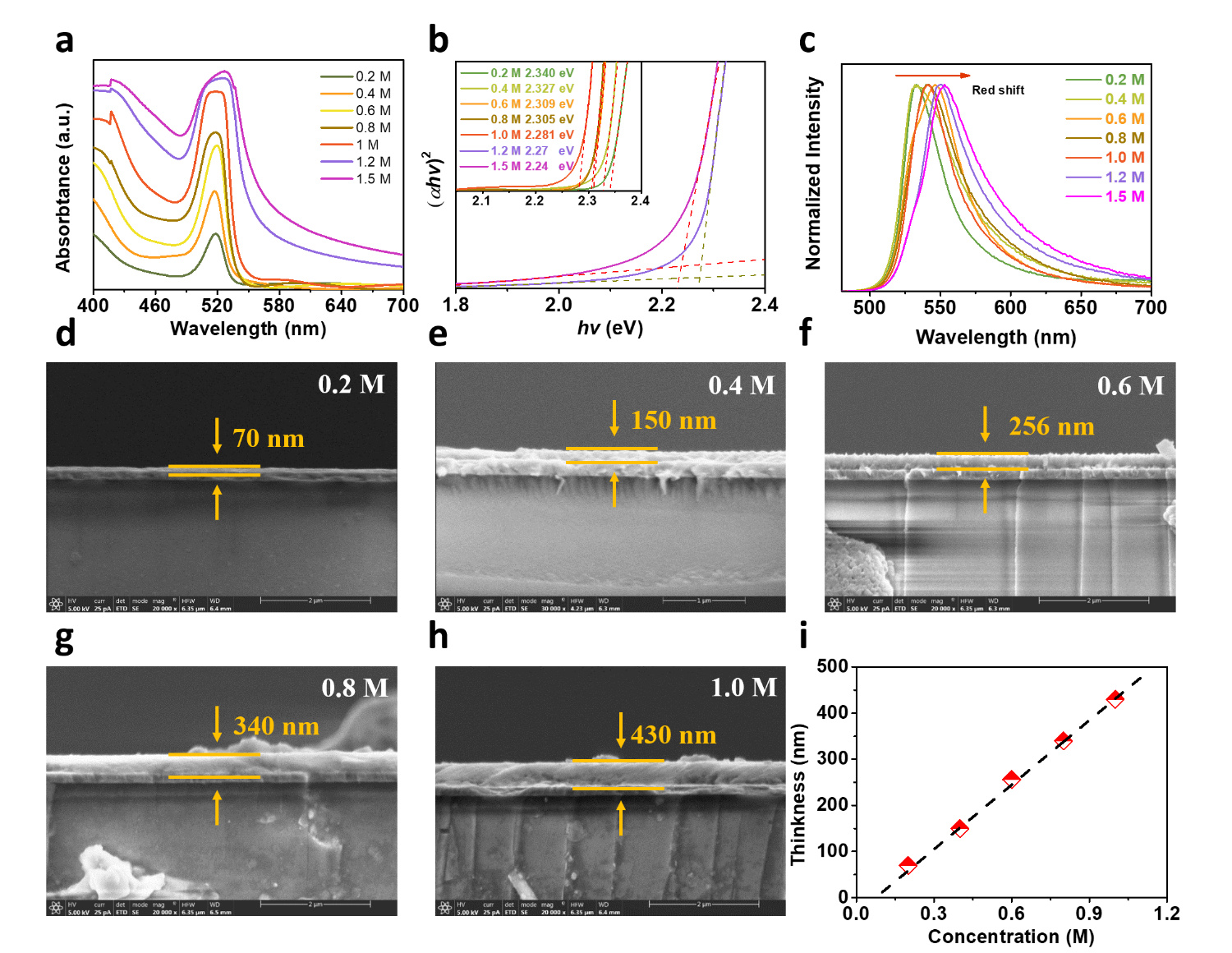


**Figure S3.** The UV-Vis, PL curves and cross-section images of (DFPD)_2_PbI_4_ with different concentration. (a) UV-Vis, (b) plot of (*αhv*)^2^ versus *hv* and (c) PL with different concentration (from 0.2 M to 1.5 M), (d), (e), (f), (g) and (h) are the cross-section image of (DFPD)_2_PbI_4_ film with precursor concentration of 0.2 M, 0.4 M, 0.6 M, 0.8 M and 1.0 M, respectively. (i) The summary of film thickness versus solution concentration (the dashed line is the fitted curve).

The absorbance of the (DFPD)_2_PbI_4_ film increases with the increase of the precursor concentration. **Figure S3a** is the UV-Vis of (DFPD)_2_PbI_4_ film with different concentration. **Figure S3b** is the bandgap calculated from the intercept of the curve plot (*αhv*)^2^ versus *hv*, as the precursor concentration increases from 0.2 M to 1.5 M, the film band gap gradually decreases from 2.34 eV to 2.24 eV. **Figure S3c** is the PL of film with different concentration, the peak position of PL red shifts with increasing concentration, which is consistent with the change of the band gap of the film.

**Figure S3d to h** are the cross-section images of different concentrations of (DFPD)_2_PbI_4_ film spin-coated on ITO glass. As the precursor concentration increases, the thickness of the film gradually increases from 70 nm to 430 nm. **Figure S3i** is the summary and fitting curve of film thickness versus precursor concentration.


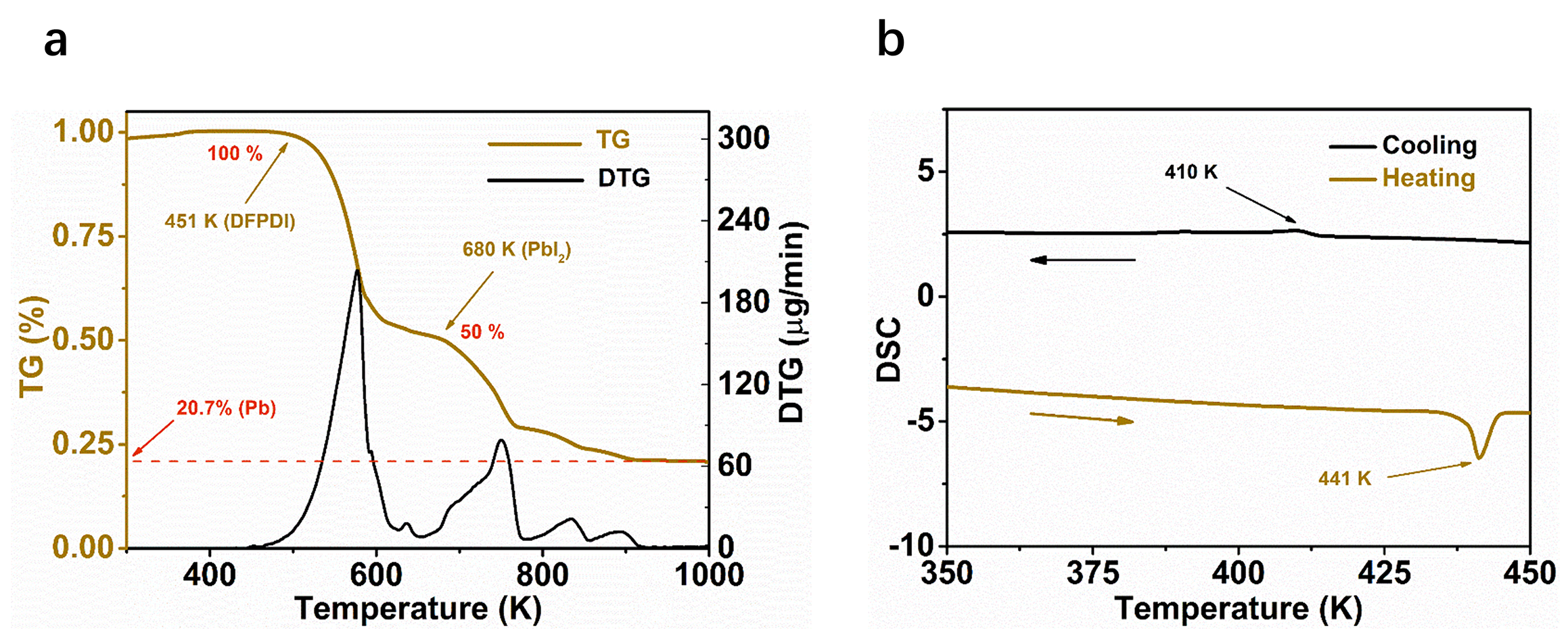


**Figure S4.** Phase transition temperature and decomposition temperature test of obtained (DFPD)_2_PbI_4_ film. (a) Thermo-gravimetric analysis (TGA) and (b) Differential scanning calorimetry (DSC).

The TGA measurement (**Figure S4a**) reveals that the obtained (DFPD)_2_PbI_4_ Polycrystalline film start to decompose at 451K, which is the loss of organic parts (4, 4-difluoropiperdinium), and the PbI_2_ is stable until 680K. The decomposition temperature is higher than the phase transition temperature, which further proves the stability of the material at room temperature. The DSC result (**Figure S4b**) shows that there is an endothermic process at 441K during the heating process, and an exothermic phenomenon occurs near 410K during the cooling process. These two endothermic and exothermic processes reveal that the phase transition temperature (Curie temperature) of the material is above room temperature, which means that the material has a stable ferroelectric phase at room temperature and can be stably polarized at room temperature.


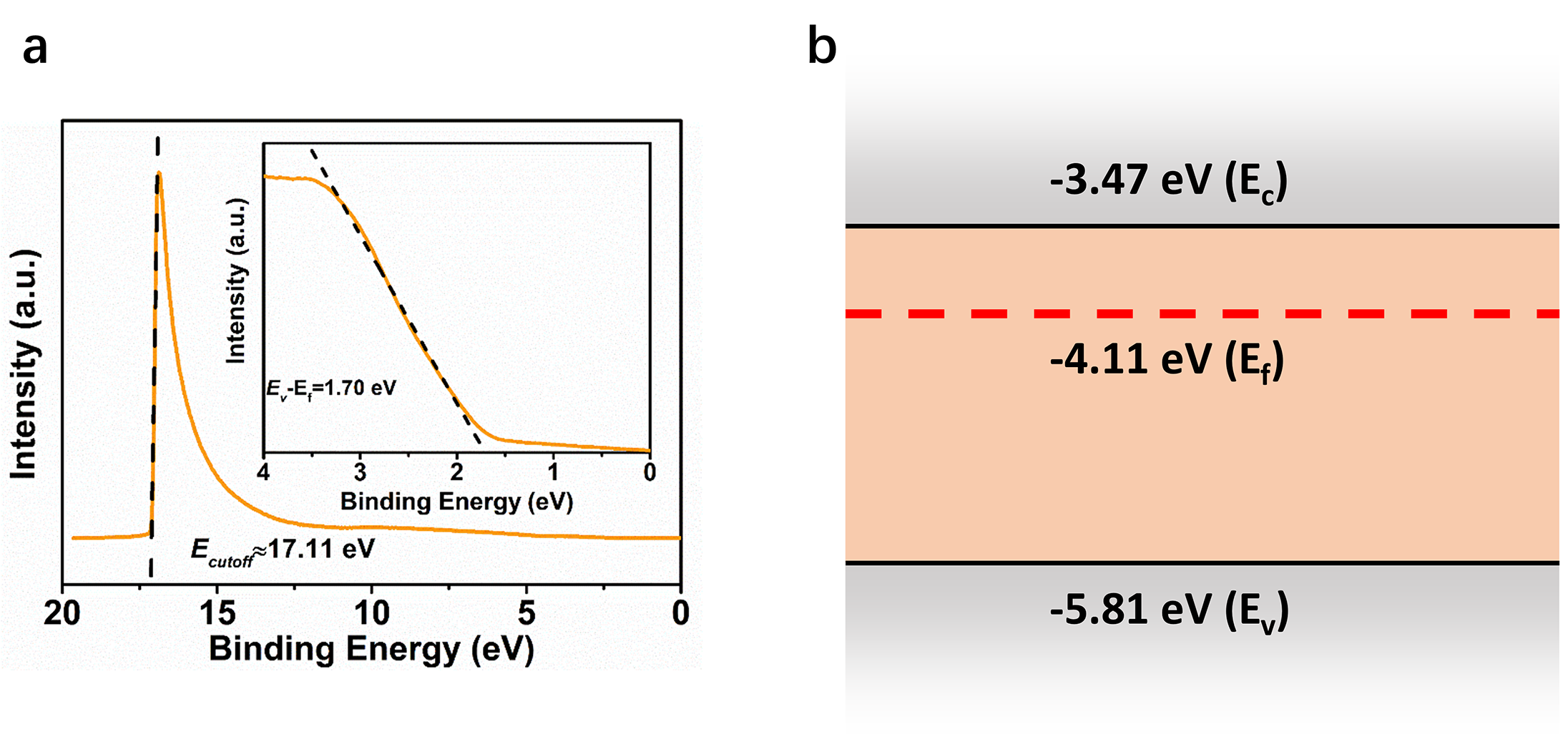


**Figure S5.** Ultraviolet Photoelectron Spectrum (UPS) of (DFPD)_2_PbI_4_ film. (a) The UPS result. (b) Band diagram.

**
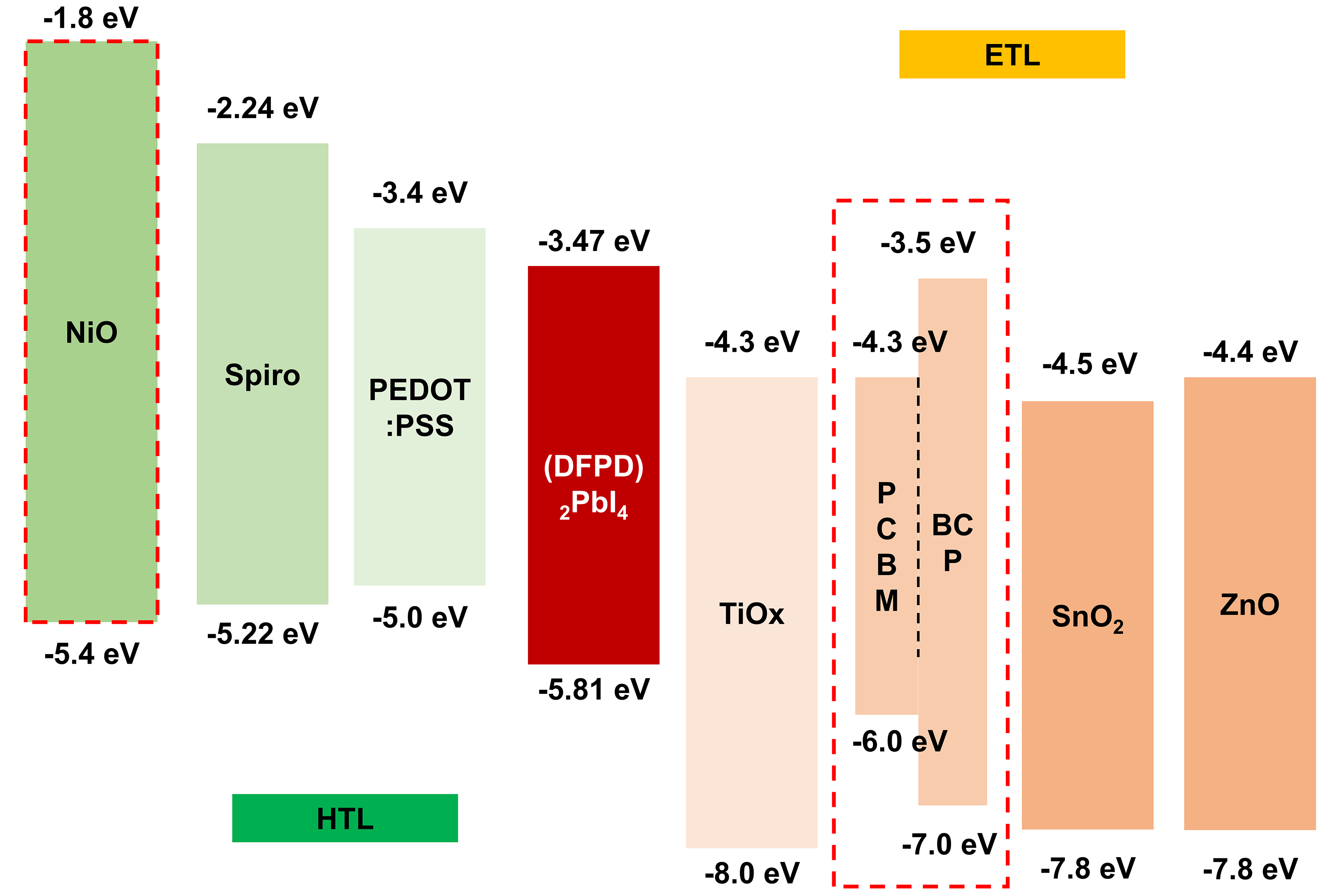
**

**Figure S6.** Band diagram of (DFPD)_2_PbI_4_ and transport layers.

Considering the inverted structure and the purpose of increasing the ability to block electrons, we use NiO_X_ with a deeper conduction band as the hole transport layer. Taking into account the material's tolerance to temperature, PCBM that does not require annealing is used as the electron transport layer. At the same time, in order to improve the ability to block holes, BCP with a deeper valence band is used as the hole blocking layer.


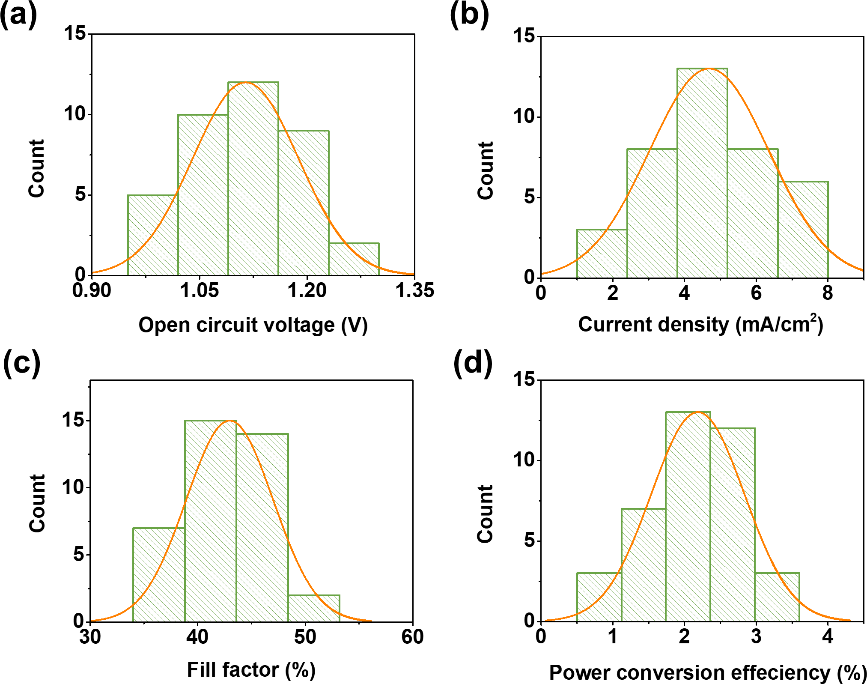


**Figure S7.** Statistics on the performance of 38 devices. (a) Open-circuit voltage, (b) Current density, (c) Fill factor and (d) Powder conversion efficiency.


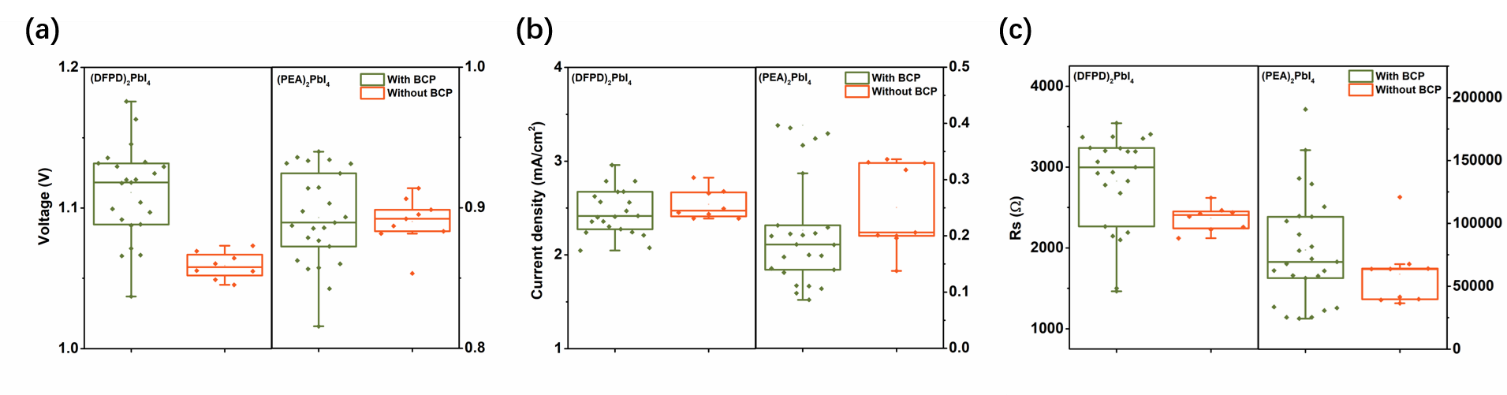


**Figure S8**. Device parameters of solar cells based on (DFPD)_2_PbI_4_ and (PEA)_2_PbI_4_. (a) Open circuit voltage (*V*_OC_), (b) current density (Rs) and (c) series resistance.

In general, bathocuproine (BCP) is adopt as a hole blocking layer, which can improve contact with electrodes and facilitate the extraction of electrons. To explore whether BCP plays a key role in improving device performance, we fabricate ferroelectric (DFPD)_2_PbI_4_ solar cells and non-ferroelectric (PEA)_2_PbI_4_ solar cells with BCP (ITO/NiO_X_/(DFPD)_2_PbI_4_/PCBM/BCP/Ag) and BCP-free (ITO/NiO_X_/(DFPD)_2_PbI_4_/PCBM/Ag). **Figure S8** show BCP play a little bit role on both of solar cells. As for non-ferroelectric (PEA)_2_PbI_4_ solar cells, there is little influence on the performance with or without BCP. As for ferroelectric (DFPD)_2_PbI_4_ solar cells, the presence of BCP in **Figure S8a** can a little bit increase the *V*_OC_ of devices. However, there is no significant difference in the short-circuit current (**Figure S8b**), which may be caused by increased series resistance (**Figure S8c**). Overall, the ferroelectric (DFPD)_2_PbI_4_ solar cells with or without BCP exhibited lower series resistance (an order of magnitude smaller) and higher current density than non-ferroelectric (PEA)_2_PbI_4_ solar cells. Therefore, BCP as a hole blocking layer indeed increase the device *V*_OC_. However, this improvement is limited, and the excellent properties of 2D ferroelectric (DFPD)_2_PbI_4_ solar cells originate from the molecular ferroelectric perovskite itself.

**Table S1.** Device parameters of 2D Ruddlesden-Popper PSCs reported in this paper and previous literatures.

| **Material** | ***V*_OC_ (V)** | ***J*_SC_ (mA/cm^2^)** | **FF (%)** | **PCE (%)** | ***E*_g_ (eV)** | **Ref.** |
| --- | --- | --- | --- | --- | --- | --- |
| (Bn)_2_SnI_4_ | 0.40 | 10.55 | 55.1 | 2.35 | 1.81 | [2] |
| (PMA)_2_CuBr_4_ | 0.68 | 0.73 | 41 | 0.2 | 1.81 | [3] |
| (*p*PDA)CuBr_4_ | 0.65 | 1.14 | 32 | 0.24 | 1.43 | [4] |
| (Anyl)_2_PbI_4_ | 0.75 | 1.20 | 74 | 0.66 | - | [5] |
| (pyrene-O-propyl-NH_3_)_2_PbI_4_ | 1.02 | 2.43 | 46 | 1.38 | 2.53 | [6] |
| (BA)_2_PbI_4_ | 0.58 | 0.06 | 29 | 0.01 | 2.24 | [7] |
| (BDA)_2_PbI_4_ | 0.87 | 2.89 | 43 | 1.08 | 2.37 | [8] |
| (HDA)_2_PbI_4_ | 0.73 | 1.74 | 47 | 0.59 | 2.44 | [8] |
| (ODA)_2_PbI_4_ | 0.73 | 0.05 | 34 | 0.01 | 2.55 | [8] |
| (F-PMA)_2_CuBr_4_ | 0.87 | 1.46 | 40 | 0.51 | 1.74 | [9] |
| (BA)_2_CuBr_4_ | 0.88 | 1.78 | 40 | 0.63 | 1.76 | [9] |
| PEA_2_PbI_4_ | 0.91 | 2.61 | 59 | 1.40 | 2.35 | [10] |
| BA_2_PbI_4_ | 0.88 | 2.56 | 57 | 1.28 | 2.36 | [10] |
| (DFPD)_2_PbI_4_ | **1.16** | 5.44 | 49.4 | **3.13** (O) | 2.32 | **This work** |
|  | **1.29** | 7.44 | 38.5 | **3.71** (P) |  |  |

(O): original 2D ferroelectric PSCs without polarization. (P): 2D ferroelectric PSCs after polarization.

These data are summarized from 2D Ruddlesden-Popper PSCs tested with sun simulator equipped with 1.5 AM filter with an irradiation intensity of 100 mW cm^-2^.


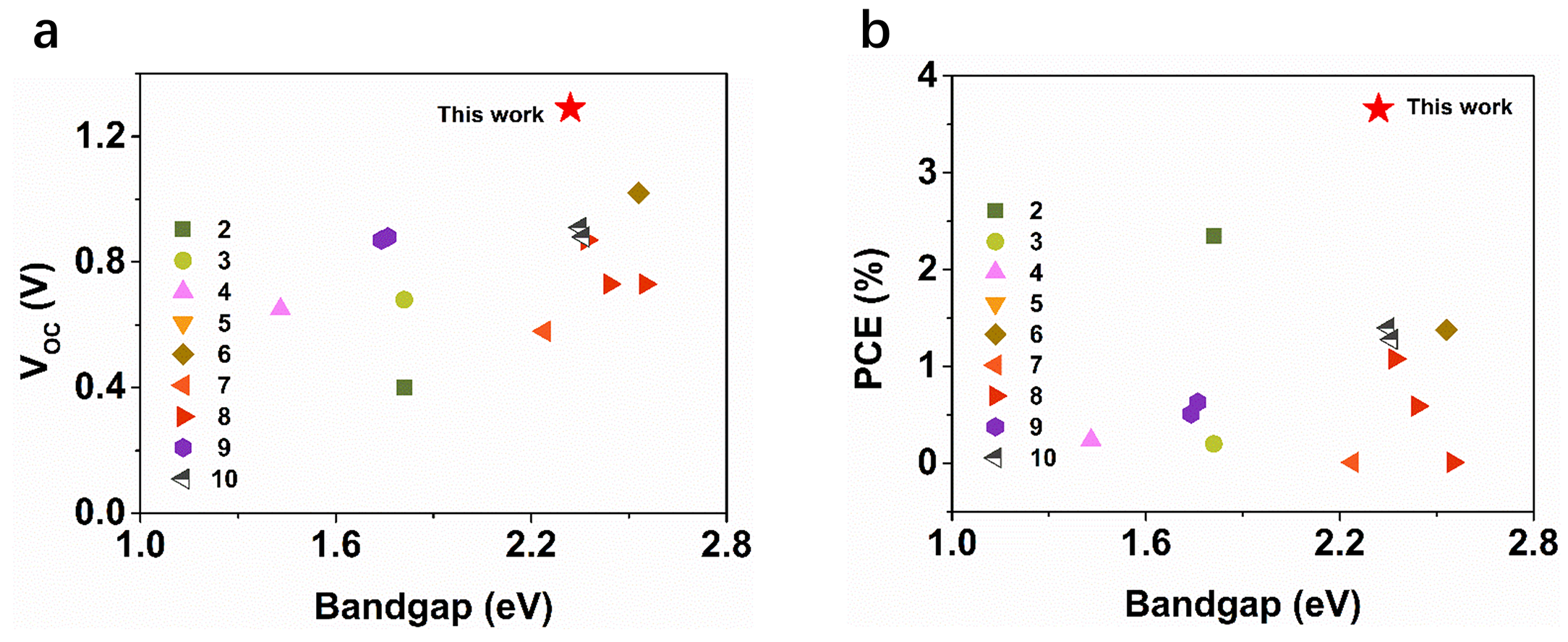


**Figure S9.** Summary of (a) *V*_OC_ versus bandgap and (b) PCE versus bandgap of 2D Ruddlesden-Popper PSCs from table S1.


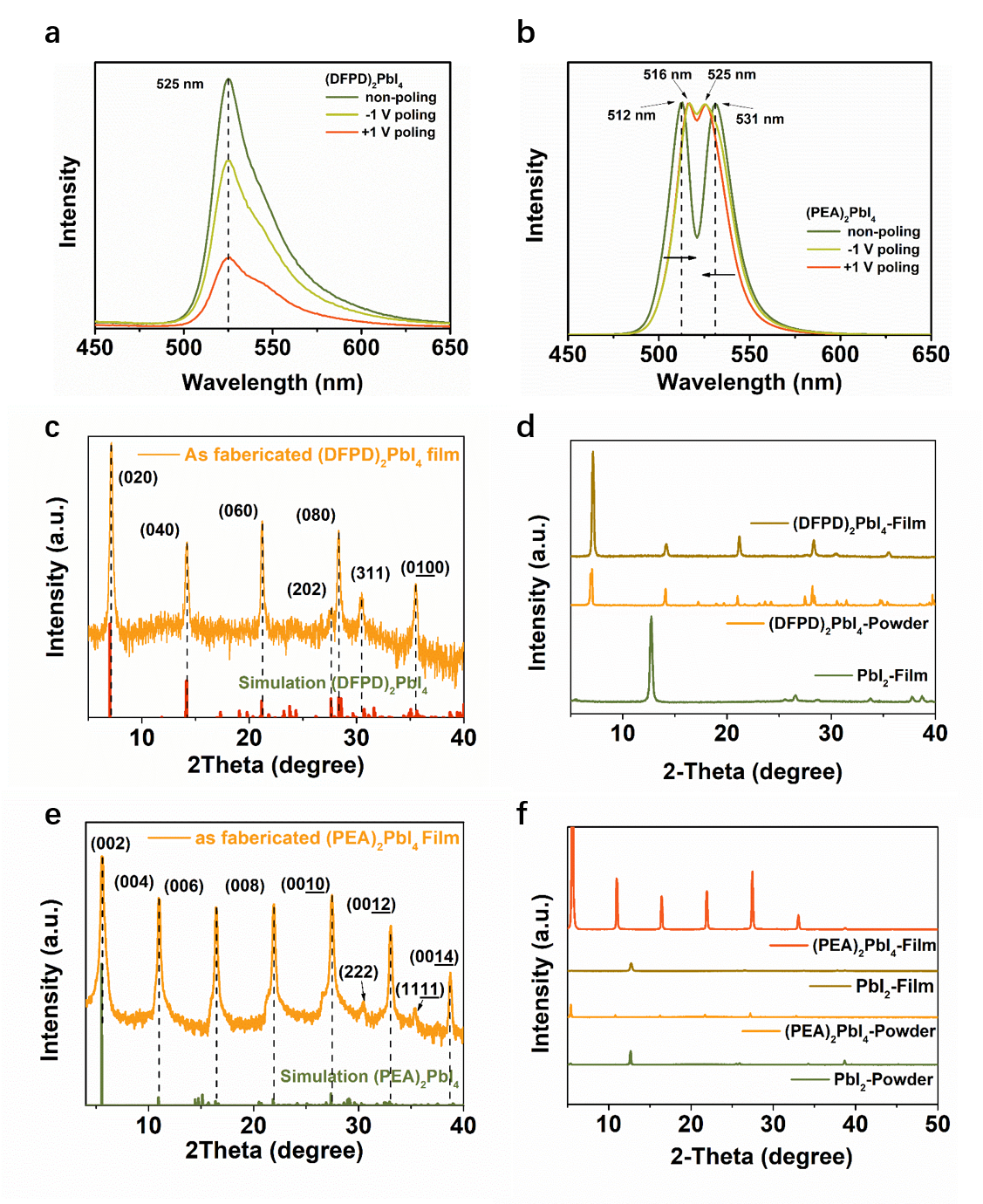


**Figure S10.** Steady-state photoluminescence (PL) of different polarization conditions. (a) (DFPD)_2_PbI_4_, (b) (PEA)_2_PbI_4_. (c) XRD of (DFPD)_2_PbI_4_ film on the log scale. (d) XRD of (DFPD)_2_PbI_4_ film, (DFPD)_2_PbI_4_ powder and PbI_2_ film. (e) XRD of (PEA)_2_PbI_4_ film on the log scale. (f) XRD of (PEA)_2_PbI_4_ film, (PEA)_2_PbI_4_ powder and PbI_2_ film.

There are asymmetric PL peaks in either as-prepared (DFPD)_2_PbI_4_ or (PEA)_2_PbI_4_ film (**Figure S10a** and **S10b**). There peaks (**Figure S10c**) match the XRD pattern of (DFPD)_2_PbI_4_ powder in CCDC 1934896 with polar space group *Aba*2 and there is no any other phase in the XRD pattern. Except for the obviously preferential-oriented (0*l*0) XRD pattern, there are (202), (311) peaks in as-grown (DFPD)_2_PbI_4_ films. To explore whether an excess of the raw material (PbI_2_) remains in as-grown film, XRD characterization on as-prepared (DFPD)_2_PbI_4_ powder (**Figure S10d**) are also carried out. Compared with the (DFPD)_2_PbI_4_ powder and pure PbI_2_, there is no PbI_2_ impurity in either the (DFPD)_2_PbI_4_ powder or the (DFPD)_2_PbI_4_ film. The XRD pattern of (PEA)_2_PbI_4_ on the log scale and XRD pattern of (PEA)_2_PbI_4_ and PbI_2_ are also carried. XRD pattern of (PEA)_2_PbI_4_ on the log scale (**Figure S10e**) reveals that no other impurity such as n>1 2D perovskite. Powder and film XRD characterization of (PEA)_2_PbI_4_ and PbI_2_ excluded the existence of excess raw material (PbI_2_) (**Figure S10f**).


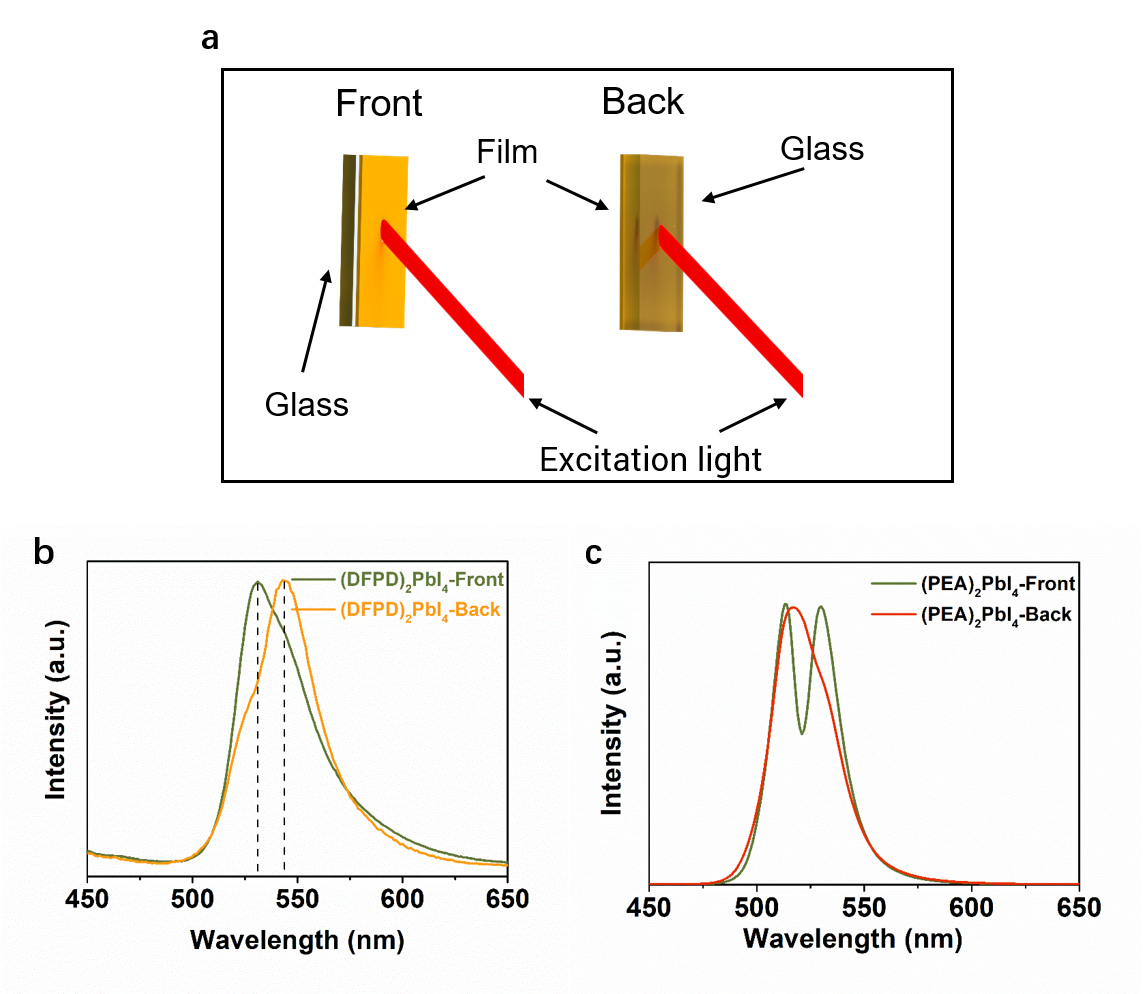


**Figure S11**. PL test excited from the front and back of the film. (a) Schematic diagram of sample excited from the front and back. (b) Dual emission peaks of (DFPD)_2_PbI_4_ film. (c) Dual emission peaks of (PEA)_2_PbI_4_.


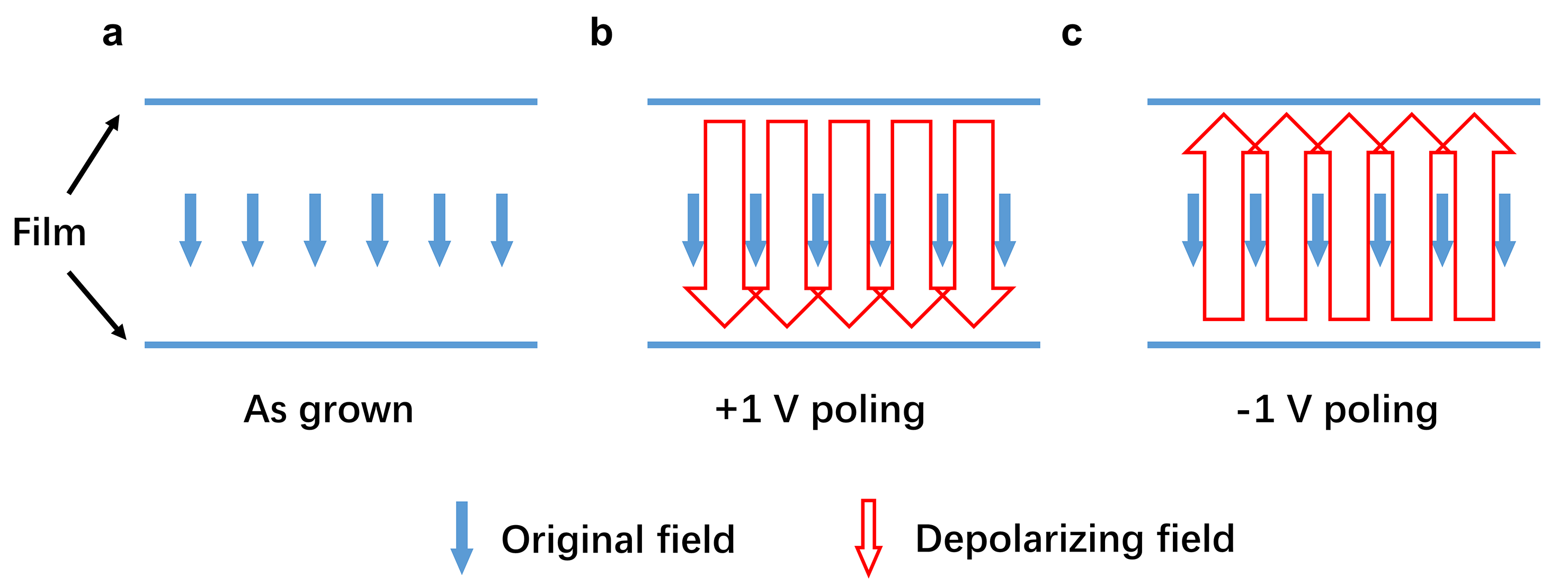


**Figure S12.** Schematic diagram of the coupling effect of spontaneous polarization field and depolarization field. (a) As grown film with spontaneous polarization, (b) The depolarization field is consistent with the original field, and the coupling field can separate the charges more effectively, leading to fluorescence quenching. (c) The direction of the depolarization field is opposite to that of the depolarization field. The existence of the spontaneous polarization field weakens the separation effect of the depolarization field on electrons and holes.

**Figure S12a** is the as-grown film with spontaneous polarization. Being polarized by +1 V, the depolarization field is the same direction as the original field (**Figure S12b**). The coupling field can more effectively separate the charges from the film to the electrode and reduce the radiative recombination, resulting in effective quenching of PL. Upon -1 V polarization, the original field with opposite orientation reduces the separation effect of the depolarization field (**Figure S12c**).


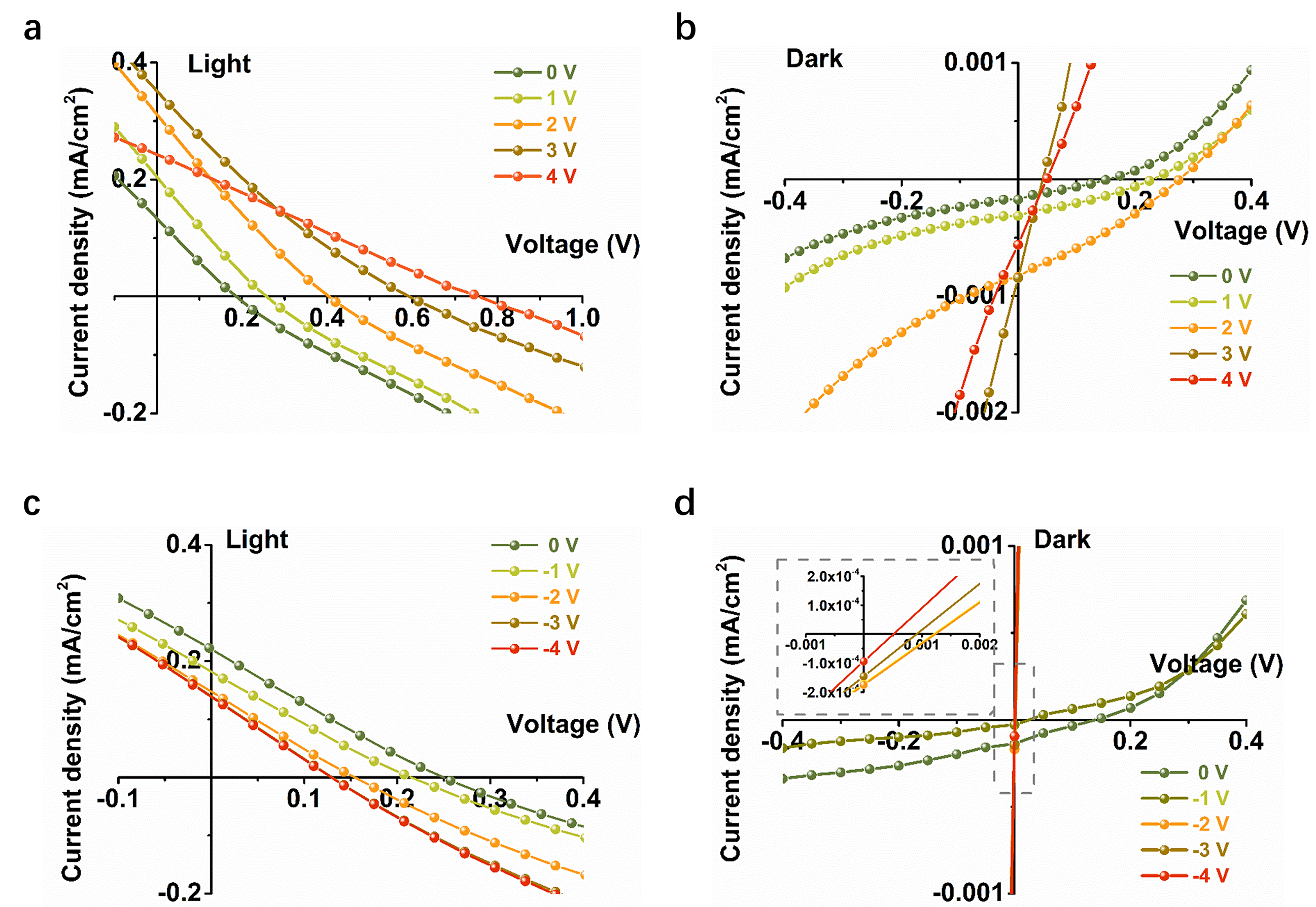


**Figure S13.** *J*-*V* curves of device based on transport layer-free device ITO/ (DFPD)_2_PbI_4_/Ag. (a) The *J*-*V* curves of the positive polarized device (from 0 V to 4 V). (b) Dark current of positive polarized device (from 0 V to 4 V). (c) The *J*-*V* curves of the reverse polarized device (from 0 V to -4 V). (d) Dark current of reverse polarized device (from 0 V to -4 V).


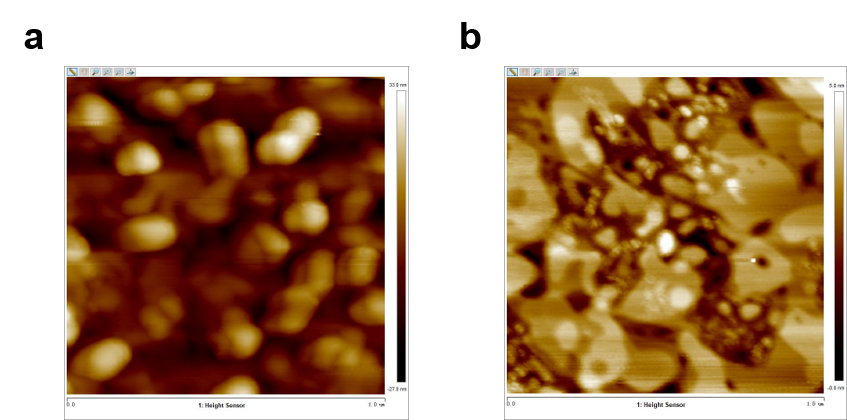


**Figure S14.** The topographic data for the PFM test. (a) Film 1, (DFPD)_2_PbI_4_, (b) Film 2, (PEA)_2_PbI_4_.


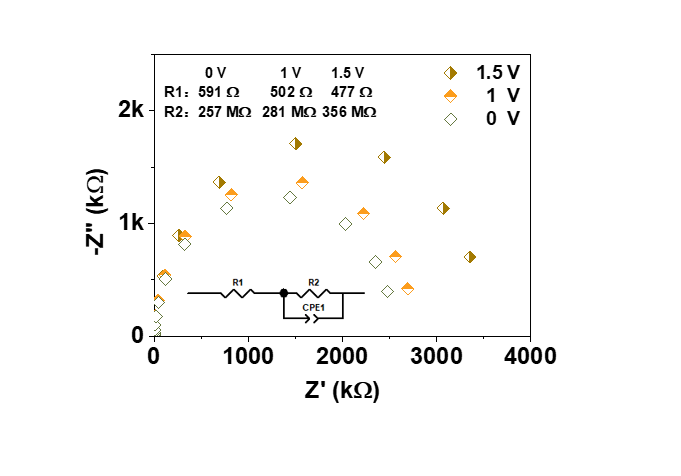


**Figure S15**. The Nyquist plots of (DFPD)_2_PbI_4_ solar cells, the inset is the equivalent circuit model.

In order to demonstrate the role of ferroelectricity on poor electron transport in MQW structures, electrochemical impedance spectroscopy analysis is performed under dark condition along with a 5 mV AC signal in the frequency range from 1 Hz to 10 KHz. The Nyquist plot of the (DFPD)_2_PbI_4_ devices are shown in **Figure S15**. The *R*1of EIS can be associated devices series resistant (*R*s). By poling with 0, 1 and 1.5 V, the *R*s reduces from 591 to 477 Ω, respectively, which means the enhanced charge transport after polarization. There is semicircle in a relatively high frequency range which indicates the charge transfer resistance and it is reasonable to associate the high-frequency semicircle (*R*2) with the total shunt resistance (*R*sh) of the solar cell.[11] Without polarization, the devices exhibit relatively low *R*_sh_ under dark field, which is due to poor electron-hole transport. After 1 V polarization, the device *R*_sh_ increases, implying less recombination in the MQW structure in coincidence with the enhanced *V*_OC_. The higher bias voltage (1.5 V) leads to a further increase in *R*_sh_. Therefore, ferroelectricity has a positive effect on weakening the potential barrier of the MQW structure and reducing the electron-hole recombination loss in 2D perovskites.

**Table S2**. Materials parameters for simulation.


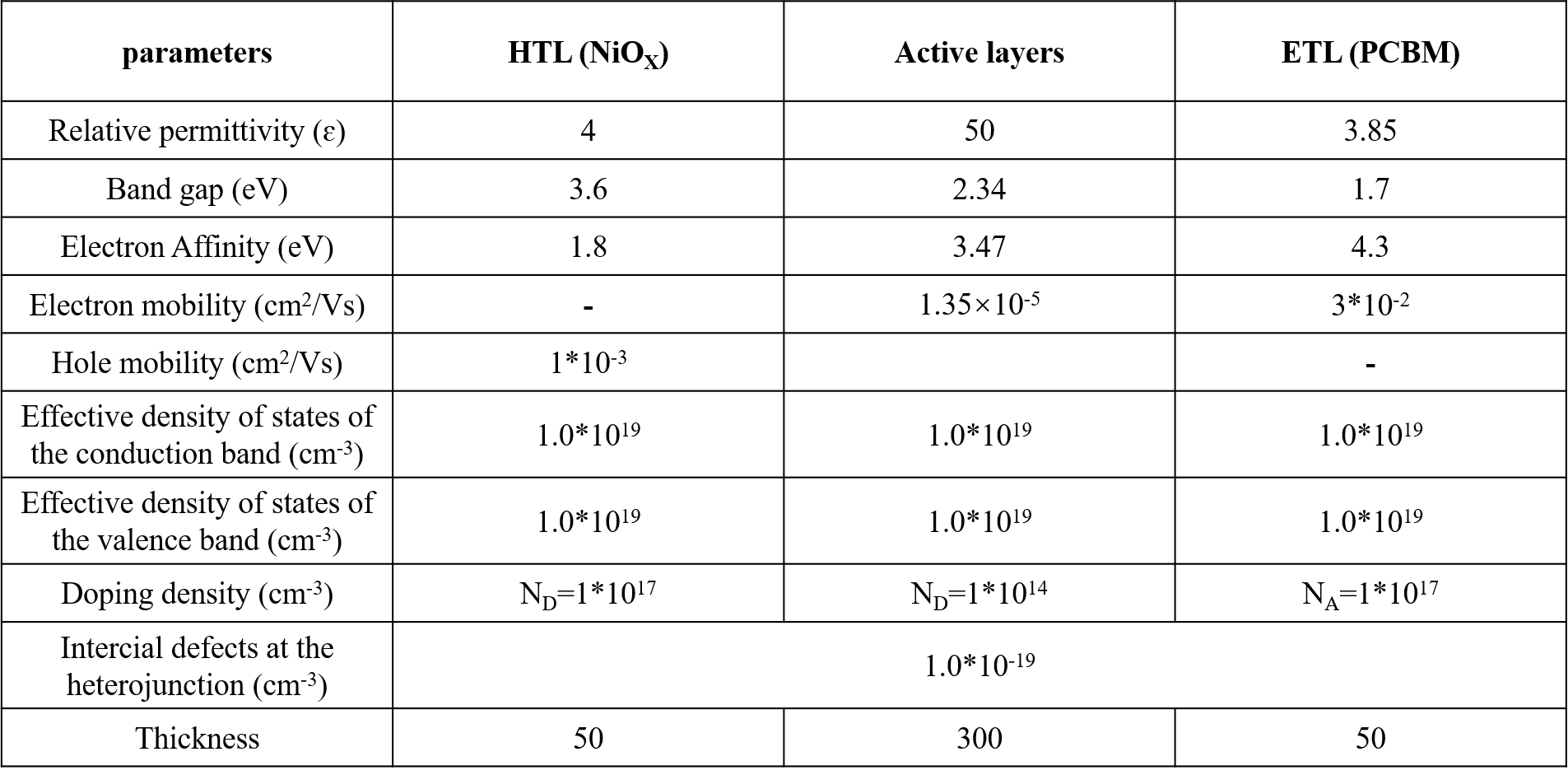


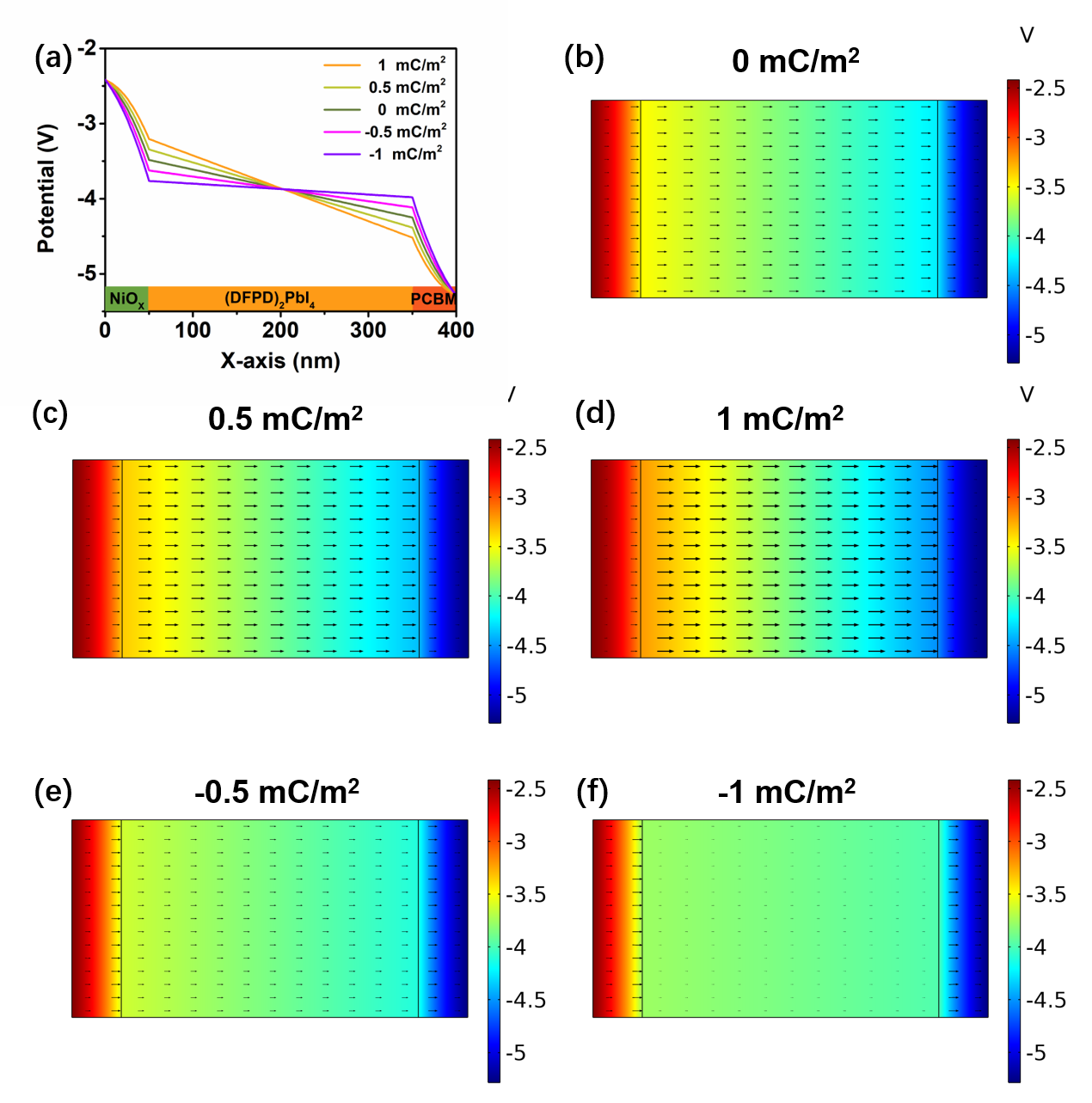


**Figure S16**. Simulation results of electron transport modulation at the heterojunction interface. (a) Electric potential distribution across the NiO_X_-(DFPD)_2_PbI_4_-PCBM with respect to the direction of electric dipoles. The electric dipoles were modulated by changing surface charge density from -1.0 to +1.0 mC/m^2^. Visualized electron flows and electric field intensity depicted by the direction and size of arrows, respectively: (b) initial state, 0 mC/m^2^, (c) positively poled with 0.5 mC/m^2^, (d) positively poled with 1 mC/m^2^, (e) negatively poled with -0.5 mC/m^2^ and (f) negatively poled with -1.0 mC/m^2^. The depolarization field strength is simulated by changing the surface charge density, and the specific values refer to P(VDF-TrFE) due to similar remanent polarization. [12]


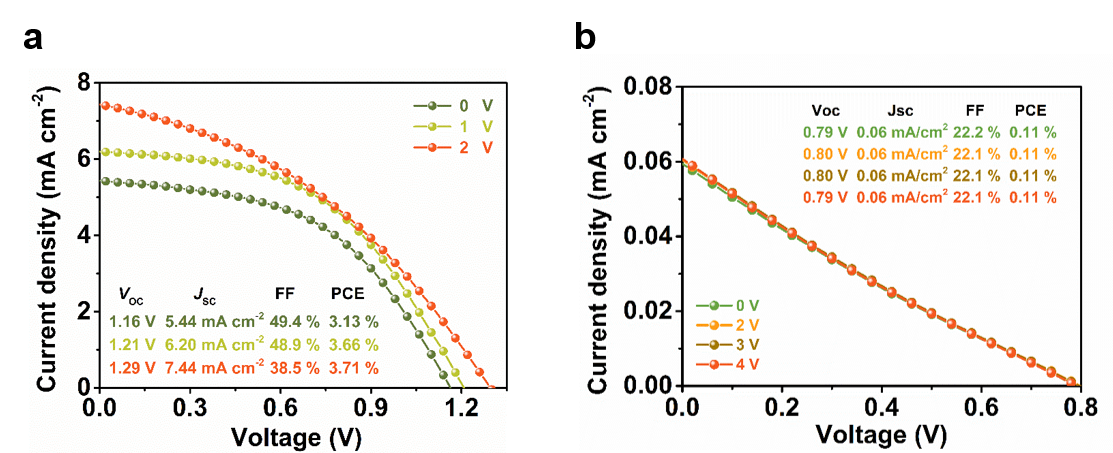


**Figure S17.** Polarization and stability characterization of 2D ferroelectric PSCs. (a) *J*-*V* curves of 2D (DFPD)_2_PbI_4_ ferroelectric PSCs and (b) 2D (PEA)_2_PbI_4_ PSCs under different bias voltage.





**Figure S18**. Field stability analysis. The *V*_mpp_ from maximum power point tracking of 2D (DFPD)_2_PbI_4_ device before and after poling with 1 V, 1.5 V, 2 V and 2.5 V, respectively.


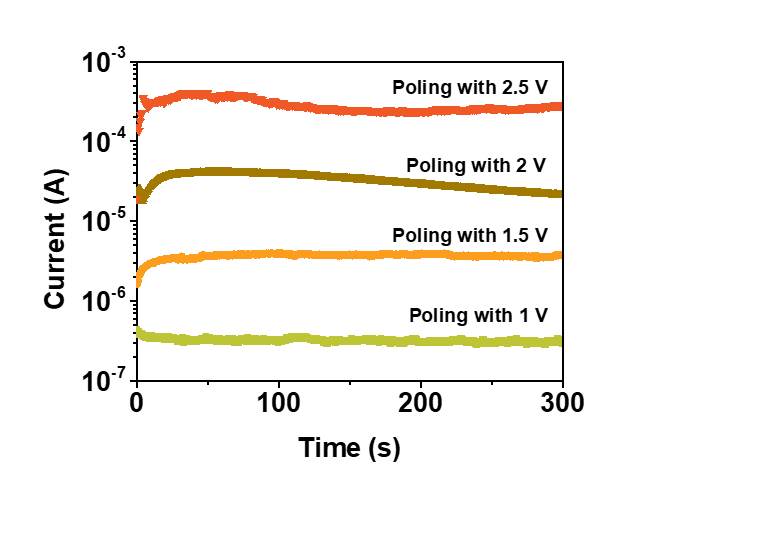


**Figure S19**. The dark current of the device during 1, 1.5, 2 and 2.5 V polarization.

In order to further discuss the irreversible damage on the device performance, the dark current of the polarized device is collected. As the polarization voltage increases from 1 V to 2.5 V, the device leakage current increases from 10^-6^ A to 10^-4^ A. It is worth noting that the leakage current of the device polarization at 2.5 V keeps increasing, which means that excessive bias voltage can result in short-circuit and device damage.

**Reference**

[1] H. Y. Zhang, X. J. Song, X. G. Chen, Z. X. Zhang, Y. M. You, Y. Y. Tang, R. G. Xiong, *J. Am. Chem. Soc.* **2020**, *142*, 4925-4931.

[2] I. Zimmermann, S. Aghazada, M. K. Nazeeruddin, *Angew Chem. Int. Ed. Engl.* **2019**, *58*, 1072-1076.

[3] X. Li, B. Li, J. Chang, B. Ding, S. Zheng, Y. Wu, J. Yang, G. Yang, X. Zhong, J. Wang, *ACS Appl. Energy Mater.* **2018**, *1*, 2709-2716.

[4] X.-L. Li, Z. Li, G. Zhang, G.-J. Yang, *J. Mater. Chem. A* **2020**, *8*, 5484-5488.

[5] J. Rodríguez-Romero, B. C. Hames, I. Mora-Seró, E. M. Barea, *ACS Energy Lett.* **2017**, *2*, 1969-1970.

[6] J. V. Passarelli, D. J. Fairfield, N. A. Sather, M. P. Hendricks, H. Sai, C. L. Stern, S. I. Stupp, *J. Am. Chem. Soc.* **2018**, *140*, 7313-7323.

[7] D. H. Cao, C. C. Stoumpos, O. K. Farha, J. T. Hupp, M. G. Kanatzidis, *J. Am. Chem. Soc.* **2015**, *137*, 7843-7850.

[8] M. Safdari, P. H. Svensson, M. T. Hoang, I. Oh, L. Kloo, J. M. Gardner, *J. Mater. Chem. A* **2016**, *4*, 15638-15646.

[9] X.-P. Cui, K.-J. Jiang, J.-H. Huang, Q.-Q. Zhang, M.-J. Su, L.-M. Yang, Y.-L. Song, X.-Q. Zhou, *Synth. Met.* **2015**, *209*, 247-250.

[10] F. Zhang, S. Y. Park, C. Yao, H. Lu, S. P. Dunfield, C. Xiao, S. Uličná, X. Zhao, L. D. Hill, X. Chen, X. Wang, L. E. Mundt, K. H. Stone, L. T. Schelhas, G. Teeter, S. Parkin, E. L. Ratcliff, Y.-L. Loo, J. J. Berry, M. C. Beard, Y. Yan, B. W. Larson, K. Zhu, *Science* **2022**, *375*, 71-76.

[11] X. L. Xu, L. B. Xiao, J. Zhao, B. K. Pan, J. Li, W. Q. Liao, R. G. Xiong, G. F. Zou, *Angew Chem. Int. Ed. Engl.* **2020**, *59*, 19974-19982.

[12] Y. Cho, B. Hou, P. Giraud, S. Pak, S. Cha, *ACS Appl. Energy Mater.* **2021**, *4*, 12056-12062.
